# Architectural engineering of Cyborg Bacteria with intracellular hydrogel^☆^

**DOI:** 10.1016/j.mtbio.2024.101226

**Published:** 2024-09-06

**Authors:** Ofelya Baghdasaryan, Jared Lee-Kin, Cheemeng Tan

**Affiliations:** Biomedical Engineering, University of California Davis, United States

**Keywords:** Cyborg Bacteria, Synthetic biology, Hydrogel, Cell division, Metabolism

## Abstract

Synthetic biology primarily uses genetic engineering to control living cells. In contrast, recent work has ushered in the architectural engineering of living cells through intracellular materials. Specifically, Cyborg Bacteria are created by incorporating synthetic PEG-based hydrogel inside cells. Cyborg Bacteria do not replicate but maintain essential cellular functions, including metabolism and protein synthesis. Thus far, Cyborg Bacteria have been engineered using one primary composition of intracellular hydrogel components. Here, we demonstrate the versatility of controlling the physical and biochemical aspects of Cyborg Bacteria using different structures of hydrogels. The intracellular cell-gel architecture is modulated using a different photoinitiator, PEG-diacrylate (PEG-DA) of different molecular weights, 4arm PEG-DA, and dsDNA-PEG. We show that the molecular weight of the PEG-DA affects the generation and metabolism of Cyborg Bacteria. In addition, we show that the hybrid dsDNA-PEG intracellular hydrogel controls protein expression levels of the Cyborg Bacteria through post-transcriptional regulation and polymerase sequestration. Our work creates a new frontier of modulating intracellular gel components to control Cyborg Bacteria function and architecture.

## Graphical abstract

**Figure:**
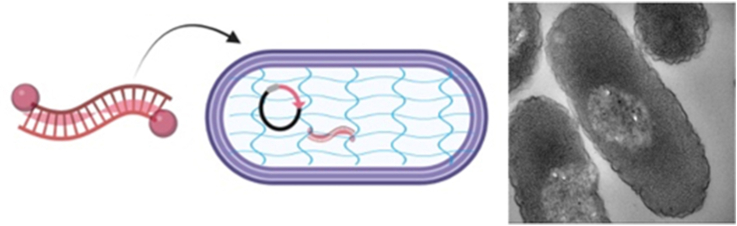

## 1 Introduction

Bridging traditional biology with engineering design, synthetic biology sets a new paradigm in the creation and modification of biological systems, reshaping the boundaries of what nature can achieve. The essence of synthetic biology lies in using computation and molecular biology tools to re-program cellular behavior [1,2]. Synthetic biology uses two primary engineering frameworks: “top-down” and “bottom-up” [3,4]. The “top-down” approach is reminiscent of fine-tuning an intricate machine of a living cell, enhancing it for specific purposes [5], be it therapeutic applications [6,7] or industrial uses [[8], [9], [10]]. Conversely, the “bottom-up” approach starts with basic, non-living elements to design and build artificial cell chassis [11,12]. Engineered live cells, although pivotal in synthetic biology, face challenges including ensuring safety and environmental containment [13,14], as well as stable genetic modifications [15]. On the other hand, artificial cells, designed to mimic natural ones, encounter challenges in replicating the complex intricate structures of natural cells [16,17]. These artificial cells often have limited functionality and stability over time, especially in diverse environments. Given the distinct challenges of engineered live cells and artificial cells, the creation of hybrid material-cell systems has very recently emerged as a promising avenue that harnesses the strengths of both systems for creating novel and robust cell chassis [3,18,19].

Specifically, Cyborg Bacteria are notable and recent examples of hybrid material-cell systems [20]. They are created by assembling and crosslinking a synthetic polymer network within each bacterium. To fabricate these Cyborg Bacteria, they are first infused with a hydrogel mixture composed of poly (ethylene glycol) diacrylate monomer (PEG-DA), 2-hydroxyl-4′-(2-hydroxyethoxy)-2-methylpropiophenone (Irgacure 2959; I2959) as the photoinitiator, and fluorescein O’O – diacrylate for fluorescence tracking. After which, ultraviolet light (UV-A) exposure triggers the crosslinking of the intracellular PEG monomers, leading to the formation of a hydrogel within the cells. This unique modification inhibits bacterial replication but maintains the integrity of their genetic material and the fluidity of their cell membrane interfaces. Notably, even with the internal hydrogel network, Cyborg Bacteria preserve essential cellular functions, including metabolism, motility, protein synthesis, compatibility with genetic circuits, and resistance to stressors otherwise lethal to natural cells. However, to fully harness the potential of the Cyborg Bacteria platform, it is essential to achieve versatile architectural control of the intracellular hydrogel for modulating functions of the Cyborg Bacteria.

**Fig. 1:**
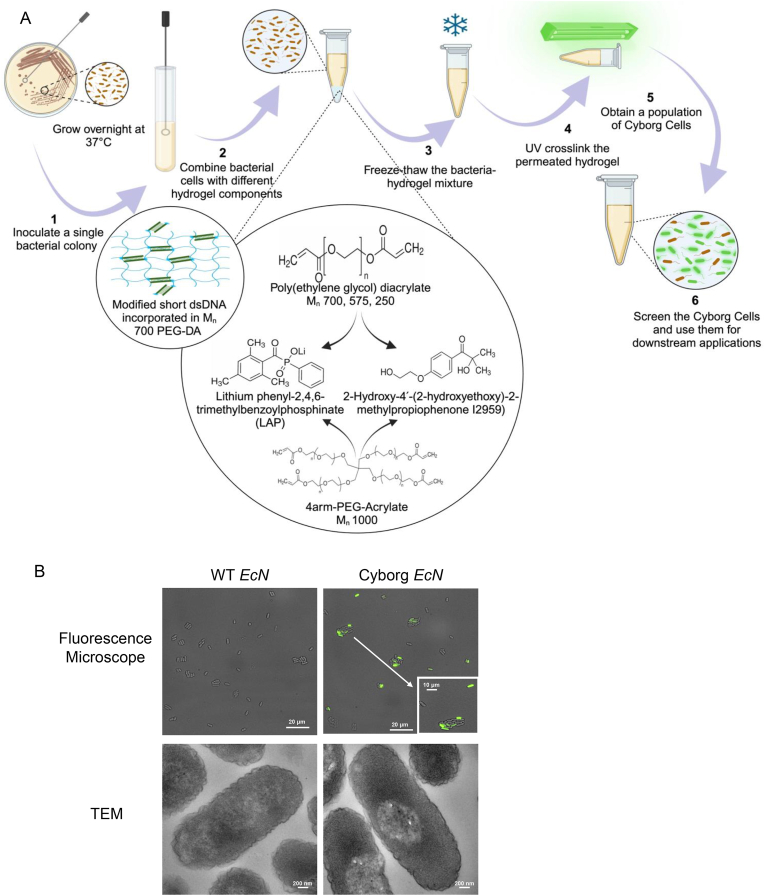
Architectural engineering of Cyborg Bacterial Cells. A) Schematic illustration of hydrogelation process and overview of the different hydrogel constituent structures. B) Fluorescence microscopy (top panel) and Transmission Electron Microscopy (TEM, bottom panel) images for WT and Cyborg EcN prepared with M_n_ 700 PEG-DA and Irgacure I2959. Cyborg Bacteria exhibit conserved bacterial cell morphology consistent with that of the WT, displaying intact cell bodies (scale bar = 20 μm top panel, 10 μm zoomed in image and 200 nm bottom panel, n = 3 biological replicates. See also Figs. S1A and S1B, S2A, S3A).

In this study, we investigated the versatility of the Cyborg Bacteria platform by altering the hydrogel composition using a new photoinitiator and poly(ethylene glycol) (PEG) variants with unique structural and molecular weight profiles (Fig. 1A and Table 1). These modifications and the resulting intracellular hydrogelation, were designed to unveil novel features and provide enhanced control over Cyborg Bacteria. Here, we substituted the photoinitiator with lithium phenyl-2, 4, 6-trimethylbenzoylphosphinate (LAP), changed the structure of PEG-DA into a branched 4-arm PEG1K-Acrylate, and varied the molecular weight (M_n_) of PEG-DA. We showed that Cyborg Bacteria maintain their non-replicating-but-metabolically-active feature. Notably, our results indicate that the permeation of the hydrogel buffer into the cells is size-restricted. Furthermore, we introduced a potential new application of Cyborg Bacteria by exploring the stability of short dsDNA crosslinked with the intracellular hydrogel. We also investigated the capability of dsDNA-PEG hydrogel to modulate mCherry protein expression levels with a 300bp DNA sequence homologous to a T7 promoter region. Our research advances Cyborg Bacteria by demonstrating the tunability of the intracellular hydrogel architecture.

**Table 1:** Table summarizing the four key tested hydrogel constituents and their unique characteristics.

LAP	Water soluble, requires less exposure to UV for crosslinking
4arm PEG1K – Acrylate	4-arms of PEG allow flexibility for conjugating any functional group for more sophisticated control of Cyborg Bacteria
M_n_ 250 and M_n_ 575 PEG-DA	PEG-DA variants with smaller molecular weight profiles to establish the intracellular hydrogelation size threshold
dsDNA (300 bases)	Short dsDNA sequence with acrydite functional groups at 5′ ends of each strand conjugated with 2-arm PEG for better stability and functionality inside the bacterial cells

## 2 Results

### 2.1 Cyborg *E. coli Nissle* 1917 can be created using a different photoinitiator

First, we established the key morphological and physiological properties of Cyborg *Escherichia coli* Nissle 1917 (EcN), our model bacterial species that is a non-pathogenic, probiotic *Escherichia coli* serotype of O6:K5:H1 [21]. Cyborg EcN was created by introducing a hydrogel buffer, comprised of poly (ethylene glycol) diacrylate monomer (PEG-DA; M_n_ 700), I2959 as a photoinitiator, and fluorescein O’O – diacrylate into EcN intracellularly following our published work [20] (Methods Section M2). Fluorescein O’O – diacrylate covalently binds with the acrylate groups on PEG-DA, serving as a fluorescent marker of the hydrogel [22].To validate and identify the resulting Cyborg Bacterial phenotype and morphology, fluorescence microscopy images of non-hydrogelated wild-type (WT) and Cyborg EcN were acquired (Fig. 1B top panel, Methods Section M6). The results showed that Cyborg EcN exhibited conserved bacterial cell morphology, i.e., rod shape and ∼1 μm length, similar to that of WT EcN cells. The effect of hydrogelation was also observed inside the cytoplasm of Cyborg EcN under TEM (transmission electron microscopy, Fig. 1B bottom right panel, Figs. S1A and S1B, Methods Section M7). Cyborg EcN exhibited denser contrasted region in the cytoplasm, as compared with WT EcN cells. Cyborg EcN had mean pixel intensity values of 28.7 (2arm-PEG/I2959), 32.1 (2arm-PEG/LAP65), 26.4 (4arm-PEG/I2959), and 64.3 (4arm-PEG/LAP65) compared to WT EcN with a mean pixel intensity value of 14.6 (Fig. S1B). These results confirm that the hydrogel infusion inside bacteria resulted in significant changes in inner cell structures.

We next investigated the efficacy of using lithium phenyl-2,4,6-trimethylbenzoylphosphinate (LAP), a widely used photoinitiator, to enable the creation of Cyborg Bacterial Cells using a less cytotoxic and faster polymerizing photoinitiator. We examined their unique physiological properties, including cell growth, hydrogelation efficiency, and metabolic activity. In the literature, LAP is favored over I2959 for biological uses because of its enhanced water solubility and faster polymerization with UV-A light. The enhanced polymerization dynamics allow for cell encapsulation using lower initiator concentrations and shorter UV-A exposure time for photocrosslinking, leading to reduced initiator toxicity and better cell survival [[23], [24], [25]]. Previous studies have demonstrated the efficacy of using different concentrations of LAP photoinitiators as photocrosslinking reagents [26].

Before creating Cyborg EcN with LAP photoinitiator-based hydrogel, we optimized the effective polymerization rate of the hydrogel buffer containing different concentrations of LAP under varying UV energy settings without cells (Methods Section M3, Fig. S2E). Our results showed that an optimal UV intensity of 800 mW/cm^2^ effectively initiated PEG-DA monomer photocrosslinking under UV. Next, we created four different hydrogel buffer compositions. Three of the buffers contained PEG-DA monomers (hereafter referred to as ‘2arm-PEG’), fluorescein O’O – diacrylate, and 24.32, 53.15, and 93.14 mM LAP in the final buffer, derived from 45, 65, and 85 mg/mL LAP solutions (Methods Section M2). The fourth buffer was prepared with I2959, adhering to our published work [20] and acted as a positive control for benchmarking the efficiency of LAP-based hydrogels. Intracellular hydrogelation of EcN with 2arm-PEG/I2959, 2arm-PEG/LAP85, 2arm-PEG/LAP65 and 2arm-PEG/LAP45 based hydrogels resulted in fluorescent green Cyborg Bacteria under a fluorescent microscope, typical of the Cyborg Bacteria phenotype (Fig. S2A). Plate reader measurement of the Cyborg Bacterial population's growth (OD _600_) and metabolic activity with a reductive metabolic dye, Presto Blue, revealed no growth in the Cyborg EcN population as compared to WT EcN cells (Fig. S2B), while the metabolic activity of Cyborg EcN cells was similar to that of WT EcN cells (Fig. S2C). Furthermore, using an ATP luminescence assay, the number of viable WT and Cyborg EcN cells in the culture was measured based on the quantification of the ATP present in each sample (Methods Section M11). Lysed WT EcN cells (as a positive control) showed detectable ATP levels, indicating that the ATP kit effectively measured ATP through the cell lysis process (Fig. S2D). In addition, carbenicillin-sorted Cyborg EcN cells showed ATP levels comparable to that of WT cells, while carbenicillin-treated WT EcN cells showed significantly reduced ATP levels than that of Cyborg Bacteria. Cyborg EcN cells also showed significantly higher levels of ATP than the heat-killed and PFA-treated EcN cells, indicating that Cyborg EcN carried out active ATP production and metabolism.

**Fig. 2:**
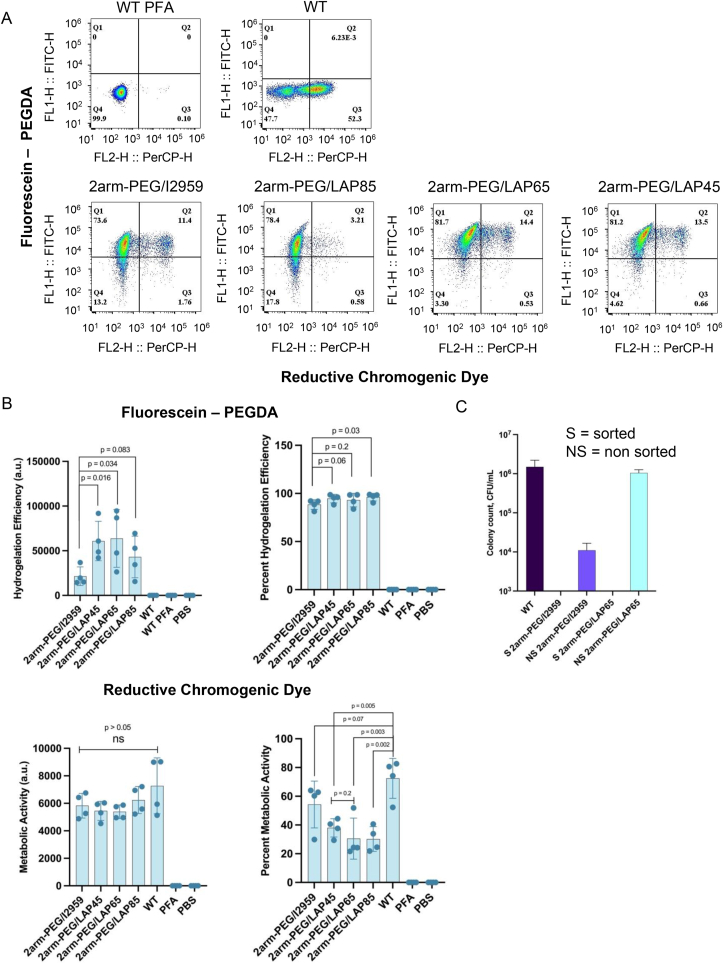
Tuning Cyborg Bacteria with LAP photoinitiator. A) Flow cytometry analysis of reductive chromogenic dye and fluorescein-PEGDA positive populations of 2arm-PEGDA coupled with I2959 and 3 different concentrations of LAP photoinitiator (Q2 = fluorescein-PEGDA and reductive chromogenic dye positive population, n = 2 biological replicates with 2 technical replicates within each biological replicate). B) (Top panels) Geometric mean and percentage of fluorescein-PEGDA positive Cyborg Bacterial populations. (Bottom panels) Geometric mean and percentage of RCD positive Cyborg Bacterial populations. The combination of 2arm-PEG/LAP65 and 2arm-PEG/LAP45 exhibited significantly higher geometric mean of fluorescein-PEGDA when contrasted with the 2arm-PEG/I2959 hydrogel combination. The mean metabolic activity of the samples was not significantly different from that of WT EcN cells, while the percent metabolic activity of 2arm-PEG/LAP formulations was significantly lower than that of WT EcN cells. No significant difference was observed between the percent RCD of Cyborg EcN formulated with 2arm-PEG/LAP65 and 2arm-PEG/LAP45 (error bar = SD, n = 2 biological replicates with 2 technical replicates within each biological replicate. See also Fig. S2B). C) CFU of sorted and non-sorted Cyborg EcN, demonstrating non detectable colony counts in sorted Cyborg Bacterial populations (error bar = SD, n = 2 technical replicates, 1.5M sorted Cyborg and WT cells, S = sorted, NS = non-sorted, detection limit at 10^3^. See also Fig. S2C).

To monitor metabolic activity on a single cell level, we used an intracellular reductive chromogenic dye (RCD), which stained the bacteria, producing a color change when the 5-cyano-2,3-ditolyl tetrazolium chloride (CTC) molecule in the dye was reduced to CTC formazan [[27], [28], [29]]. Flow cytometry analysis was performed according to the described methods (Methods Section M4) and quantified and compared through geometric means and percentages. In flow plots of bacterial cells, geometric means summarize the central tendency of the log-normally distributed datasets, which is useful for sets of positive and exponentially distributed data. The geometric mean provides an average measure of fluorescence intensity, which indicates the level of fluorescence expression among the positive cells and provides an interpretation of how effective the positive cells are at expressing the fluorescent dye. On the other hand, percentage values derived from the flow cytometry plots reflect the percentages of the total bacterial population that are hydrogelated and metabolically active. In comparison to the geometric mean values, the percentages reflect the number of cells that are hydrogelated and active. As a baseline, flow cytometry with the green fluorescent reporter fluorescein indicated that our Cyborg EcN was intracellularly gelated. WT EcN cells, as a negative control for intracellular hydrogelation, did not display any fluorescein – PEGDA signal, while ∼50 % of the population displayed metabolic activity (Fig. 2A, region Q3). Quantified geometric means of fluorescein and RCD (Fig. 2A, region Q2) revealed that LAP-tuned Cyborg EcN, particularly with 2arm-PEG/LAP65 and 2arm-PEG/LAP45, demonstrated significantly higher hydrogelation efficiency compared to 2arm-PEG/I2959 (Fig. 2B, top panel). The percentage of fluorescein-positive populations (Fig. 2A, Q1 + Q2) showed no significant difference in hydrogelation efficiency between Cyborg EcN tuned with 2arm-PEG/LAP65 or 2arm-PEG/LAP45 when compared to 2arm-PEG/I2959, while 2arm-PEG/LAP85 had significantly higher efficiency than 2arm-PEG/I2959. The percentage of RCD and RCD geometric mean varied. Comparing the geometric means of metabolic activity, no significant difference was observed between each Cyborg EcN group and WT EcN cells (Fig. 2B, bottom panel). With percent metabolic activity (Fig. 2A, Q2 + Q3), only 2arm-PEG/I2959 tuned Cyborg EcN showed the same level of metabolic activity compared to WT cells, while all other groups showed significantly reduced activity. No significant difference in metabolic activity was observed between Cyborg EcN formed with 2arm-PEG/LAP65 and 2arm-PEG/LAP45. These findings confirm that the Cyborg Bacterial Platform is tunable with LAP based photoinitiator, generating Cyborg Bacteria with higher hydrogelation efficiency and not significantly different metabolic activity from WT EcN cells. LAP65 and LAP45 showed comparable results in terms of RCD and fluorescein hydrogelation efficiency with no significant difference between the populations. We used LAP65 for all subsequent experiments. This choice provided a standard to optimize the protocol and maintain the consistency of experiments.

To further characterize the phenotype of WT and Cyborg EcN tuned with I2959 and LAP-based hydrogels, we measured the colony forming unit (CFU) with fluorescence-activated cell sorted (FACS) Cyborg Bacterial populations (Methods Section M5). Given that Cyborg Bacteria ceased replicating after gelation, the CFU assay confirmed that FACS-sorted (S) 2arm-PEG/I2959 tuned Cyborg Bacteria did not yield any detectable colonies (consistent with our established protocol). Similarly, FACS-sorted 2arm-PEG/LAP65 did not yield any detectable colonies when compared with that of WT EcN cells (Fig. 2C). In comparison, the non-sorted (NS) 2arm-PEG/I2959 and 2arm-PEG/LAP65 populations exhibited roughly 2-log-fold and 0.2-log-fold reductions in colony counts when compared with WT EcN cells, respectively. In summary, our results confirm that by using the LAP photoinitiator, we can fine-tune Cyborg Bacteria to create a metabolically active population that is non-dividing.

### 2.2 Cyborg *E. coli Nissle* can be tuned with distinct architectural hydrogel variants

To engineer Cyborg Bacteria with a structurally distinct hydrogel, we investigated 4arm-PEG Acrylate M_n_ 1000 (henceforth “4arm-PEG”) to expand the stability and activity of cells through modifying density, complex network formation, and enhanced mechanical properties compared to linear 2arm-PEGDA hydrogels [30]. Unlike M_n_ 700 PEG-DA, which has two acrylate functional groups at each end of the PEG molecule, creating a linear structure, 4arm-PEG Acrylate features four acrylate groups branching from a central core. This structure potentially allows for denser hydrogel networks than M_n_700 PEG-DA [[31], [32], [33], [34]]. We prepared two hydrogel buffers: one with 4arm-PEG Acrylate and I2959 (4arm-PEG/I2959); and the other with 4arm PEG Acrylate and LAP65 (4arm-PEG/LAP65) (Methods Section M2). Intracellular hydrogelation of EcN with 4arm-PEG/I2959 and 4arm-PEG/LAP65, resulted in fluorescent green Cyborg Bacteria under a fluorescent microscope, typical of the Cyborg Bacterial phenotype (Fig. S3A).

**Fig. 3:**
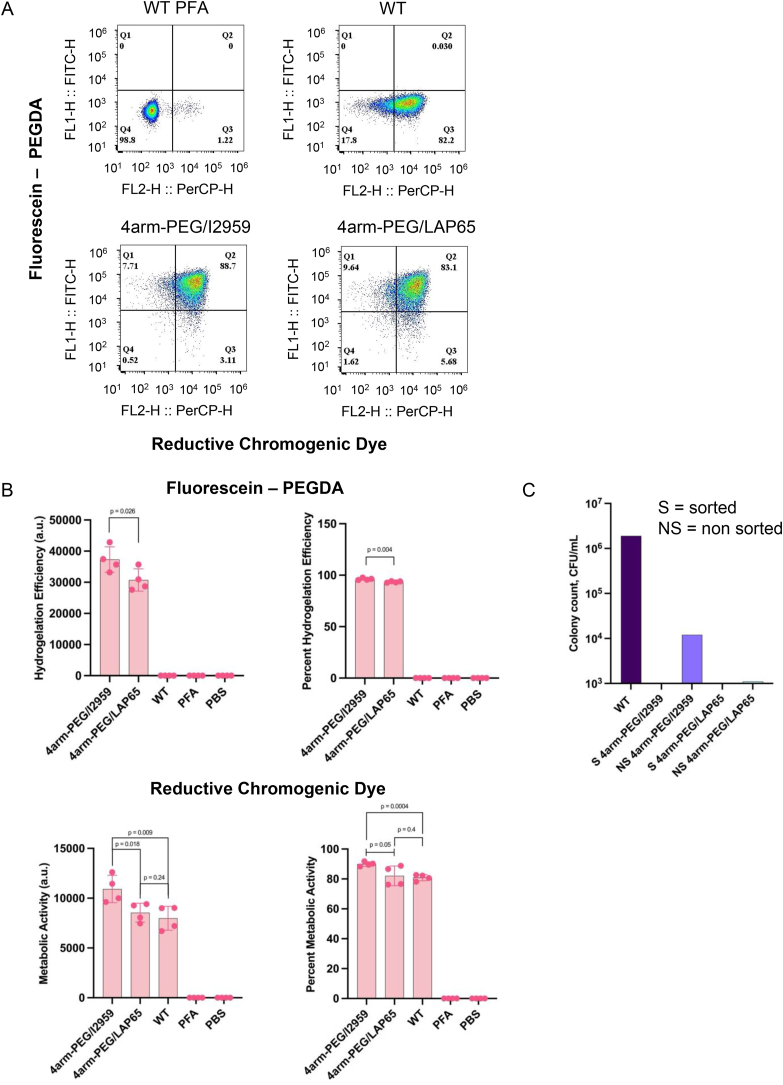

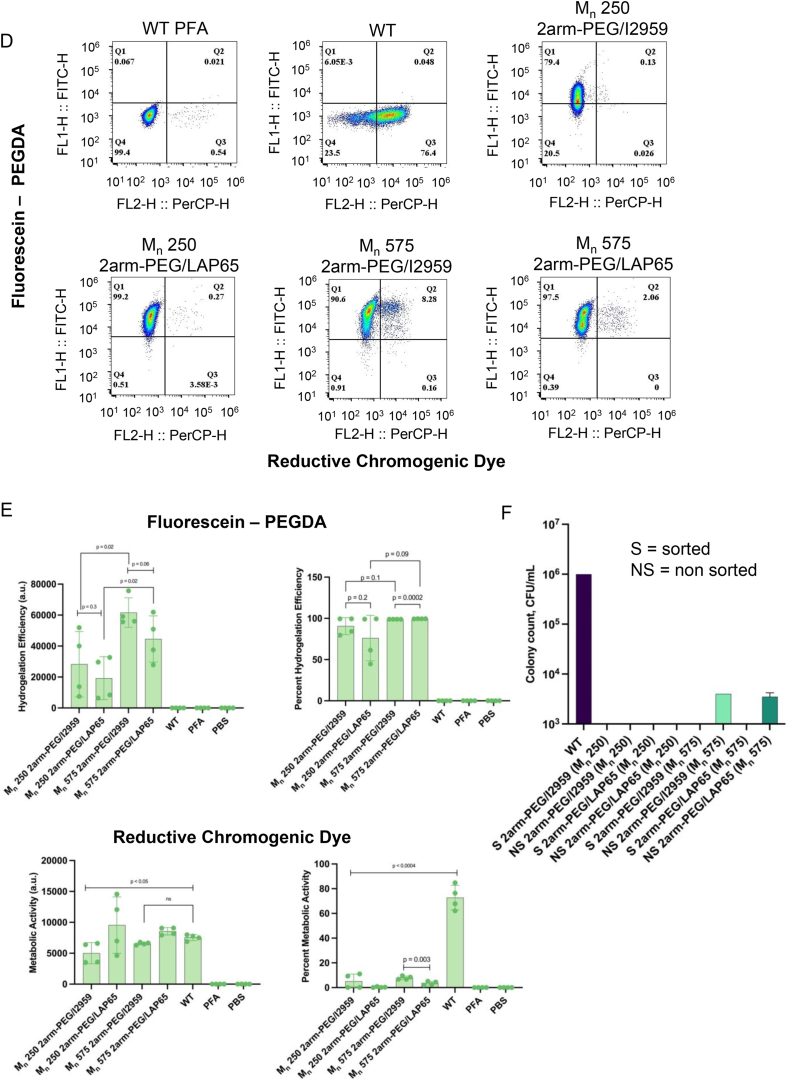
Tuning Cyborg Bacteria through Poly(ethylene glycol) variants with distinct structural and molecular weight profiles. A) Flow cytometry analysis of reductive chromogenic dye and fluorescein-PEGDA positive populations of 4arm-PEGDA coupled with I2959 and LAP65 photoinitiators (Q2 = fluorescein-PEGDA and reductive chromogenic dye positive population, n = 2 biological replicates with 2 technical replicates within each biological replicate). B) (Top panels) Geometric mean and percentage of fluorescein-PEGDA positive Cyborg Bacterial populations. (Bottom panels) Geometric mean and percentage of RCD positive Cyborg Bacterial populations. Significantly reduced mean and percent hydrogelation efficiency was observed between 4arm-PEG/I2959 and 4arm-PEG/LAP65 tuned Cyborg EcN populations, yet both groups still showed >90 % hydrogelation yield. Percent and mean metabolic activities were not significantly different between Cyborg EcN tuned with 4arm-PEG/LAP65 and WT EcN, while mean metabolic activity of 4arm-PEG/I2959 was significantly higher than that of WT EcN cells (error bar = SD, n = 2 biological replicates with 2 technical replicates within each biological replicate). C) CFU of sorted and non-sorted Cyborg *E. coli* Nissle cells, demonstrating non detectable colony counts in sorted Cyborg Bacterial populations (1.5M sorted Cyborg and WT cells, S = sorted, NS = non-sorted, detection limit at 10^3^). D) Flow cytometry analysis of reductive chromogenic dye and fluorescein-PEGDA positive populations of M_n_ 250 and 575 2arm-PEGDA coupled with I2959 and LAP65 photoinitiators (Q2 = fluorescein-PEGDA and reductive chromogenic dye positive population, n = 2 biological replicates with 2 technical replicates within each biological replicate). E) (Top panels) Geometric mean and percentage of fluorescein-PEGDA positive Cyborg Bacterial populations. (Bottom panels) Geometric mean and percentage of RCD-positive Cyborg Bacterial populations. Results indicate no significant difference in percent positive fluorescein diacrylate bacteria in M_n_ 250 and 575 2arm-PEGDA Cyborg Bacteria with only slight increase (6.22 %) in percent positive RCD bacteria in 575 2arm-PEGDA with I2959 compared to LAP65. There was a notable increase in hydrogelation efficiency in bacteria with M_n_ 575 2arm-PEGDA compared to M_n_ 250 2arm-PEGDA in both I2959 and LAP65 (error bar = SD, n = 2 biological replicates with 2 technical replicates within each biological replicate). F) CFU of sorted and non-sorted Cyborg EcN, demonstrating three-log-fold reduction in colony counts between WT and sorted Cyborg Bacteria (error bar = SD, n = 2 technical replicates, 1.5M sorted Cyborg and WT cells, S = sorted, NS = non-sorted, detection limit at 10^3^).

We first investigated the activity and gelation level of the resulting Cyborg Bacteria through flow cytometry (Fig. 3A). The geometric mean and percentage of fluorescein were significantly different between 4arm-PEG/I2959 and 4arm-PEG/LAP65 tuned Cyborg EcN. 4arm-PEG/LAP65 tuned Cyborg EcN showed slightly reduced hydrogelation efficiency, yet both populations had over 90 % hydrogelated bacterial cells (Fig. 3B, top panel). The geometric mean and percentage of RCD showed that Cyborg EcN tuned with 4arm-PEG/LAP65-based hydrogel had comparable metabolic activity to WT EcN cells, while Cyborg EcN tuned with 4arm-PEG/I2959 showed significantly higher metabolic activity as compared with that of WT EcN cells (Fig. 3B, bottom panel). No significant difference was observed between the percent RCD of Cyborg EcN tuned with 4arm-PEG/I2959 and 4arm-PEG/LAP65 samples. In contrast to the RCD geometric means of 2arm-PEG/I2959 and 2arm-PEG/LAP65 Cyborg EcN (from Fig. 2B), the 4arm-PEG variants exhibited approximately 2-fold and 1.5-fold increases in RCD geometric mean for 4arm-PEG/I2959 and 4arm-PEG/LAP65, respectively (Fig. S3B, bottom panel). The fluorescein geometric mean in 4arm-PEG/I2959 Cyborg EcN was approximately 1.5-fold higher than the fluorescein geometric mean in 2arm-PEG/I2959 tuned Cyborg EcN (Fig. S3B, top panel). In summary, our results demonstrate that structurally branched variants of PEG can be used to tune Cyborg Bacteria and achieve comparable non-dividing-but-active characteristics of Cyborg Bacteria. Furthermore, intracellular hydrogelation with the branched polymer 4arm-PEG generated metabolic active populations of Cyborg Bacteria that surpass those tuned with M_n_ 700 PEG-DA. Notably, we tested an intracellular hydrogelation of Cyborg EcN cells with a larger 4arm-PEG Acrylate variant with a molecular weight of 2000 Da, and our results showed that the 4arm-PEG Acrylate M_n_ 2000 based hydrogel could not permeate into the cytoplasm of EcN cells and caused bacterial cell lysis during the gelation process due to its exerted toxicity over the EcN cells (Fig. S4) [35]. Thus, we confirmed that bacterial intracellular hydrogelation is dependent on the molecular weight of the introduced hydrogel, and above M_n_ 1000, intracellular hydrogelation could not be carried out.

We then investigated the cell-division phenotype of the 4arm-PEG-Cyborg Bacteria. FACS sorted colony counts of WT and Cyborg EcN tuned with either 4arm-PEG/I2959 or 4arm-PEG/LAP65 corroborated that, irrespective of the hydrogel tuning, Cyborg Bacteria did not produce detectable colonies. Conversely, the non-sorted Cyborg Bacterial populations exhibited a significant reduction in colony formation, with approximately 2-log and 3-log fold decreases for 4arm-PEG/I2959 and 4arm-PEG/LAP65, respectively, when compared to WT EcN cells (Fig. 3C). We, therefore, established that these populations are rendered non-dividing post-sorting, which is an expected trait of Cyborg Bacteria.

We next varied the molecular weight of PEG monomers between M_n_ 575 2arm-PEG and M_n_ 250 2arm-PEG, effectively changing the porosity of intracellular hydrogels [36]. Prior work has shown that PEG-based hydrogelation with M_n_ 700 2arm-PEGDA has pore sizes ranging from 100 nm to 5 μm, and incorporation of larger molecular weight PEG-DA (M_n_ 300,000) can increase pore size at the micron-scale [37], suggesting that modulation of PEGDA's molecular weight could change the structure of the intracellular hydrogel [38]. Furthermore, hydrogels of reduced pore size could restrict the diffusion of small molecules [39]. Inside Cyborg Bacteria, we assume that the reduced pore size would restrict protein movement and, therefore, reduce cell activity (reported by RCD). Flow cytometry analysis showed that hydrogelation with both M_n_ 250 2arm-PEG and M_n_ 575 2arm-PEG did not significantly change the percent of fluorescein-positive cells (Fig. 3D, Q1 + Q2). Furthermore, when crosslinked with LAP65, RCD activity decreased by approximately 76.13 % for the M_n_ 250 2arm-PEG Cyborg Bacteria and 74.34 % for the M_n_ 575 2arm-PEG Cyborg compared to WT cells (Fig. 3A and D, Q2). Notably, when crosslinked with the I2959 and compared to LAP65, RCD activity increased 6.22 % in M_n_ 575 2arm-PEG Cyborg Bacteria and showed no significant change in the M_n_ 250 2arm-PEG Cyborg Bacteria (Fig. 3E). Crosslinking with either LAP65 or I2959 showed a significant decrease in RCD in both M_n_ 250 and M_n_ 575 2arm-PEG Cyborg Bacteria compared to WT cells. While there was no difference in RCD geometric means between the M_n_ 250 and M_n_ 575 2arm-PEG Cyborg Bacteria, there was a significant increase in fluorescein geometric means of M_n_ 575-hydrogelated Cyborg Bacteria compared to those hydrogelated with M_n_ 250 2arm-PEG (Fig. 3E). These results support that the adjustment of intracellular hydrogels can influence cell activity, and reduction of PEG-length is directly associated with decreased RCD, implying diminished reductive ability of the Cyborg Bacteria.

Next, we assessed the replication ability of Cyborg EcN under the varying molecular weights and photoinitiators. Cyborg Bacteria were either directly used for agar plating after hydrogelation (NS) or isolated using FACS sorting (S) and then agar plated (Methods Section M5). The CFU results revealed minimal growth in the non-sorted M_n_ 575 2arm-PEG/LAP65 sample (approximately 3500 CFU) and the M_n_ 575 2arm-PEG/I2959 sample (approximately 4000 CFU). After cell sorting of Cyborg populations based on only fluorescein intensity, CFU was not detected in either M_n_ 575 or M_n_ 250 2arm-PEG-treated Cyborg EcN (Fig. 3F). These findings substantiate the impact of intracellular hydrogelation on bacterial cell states, resulting in non-replicating-but-active Cyborg EcN, and confirm that this method of intracellular hydrogelation produces minimal variation regardless of gel concentrations. This additionally establishes the approximate maximum (M_n_ 1000) and minimum (M_n_ 700) molecular weight PEG hydrogels for optimum cell activity.

### 2.3 Stability and functionality of short dsDNA crosslinked hydrogels in Cyborg Bacteria

**Fig. 4:**
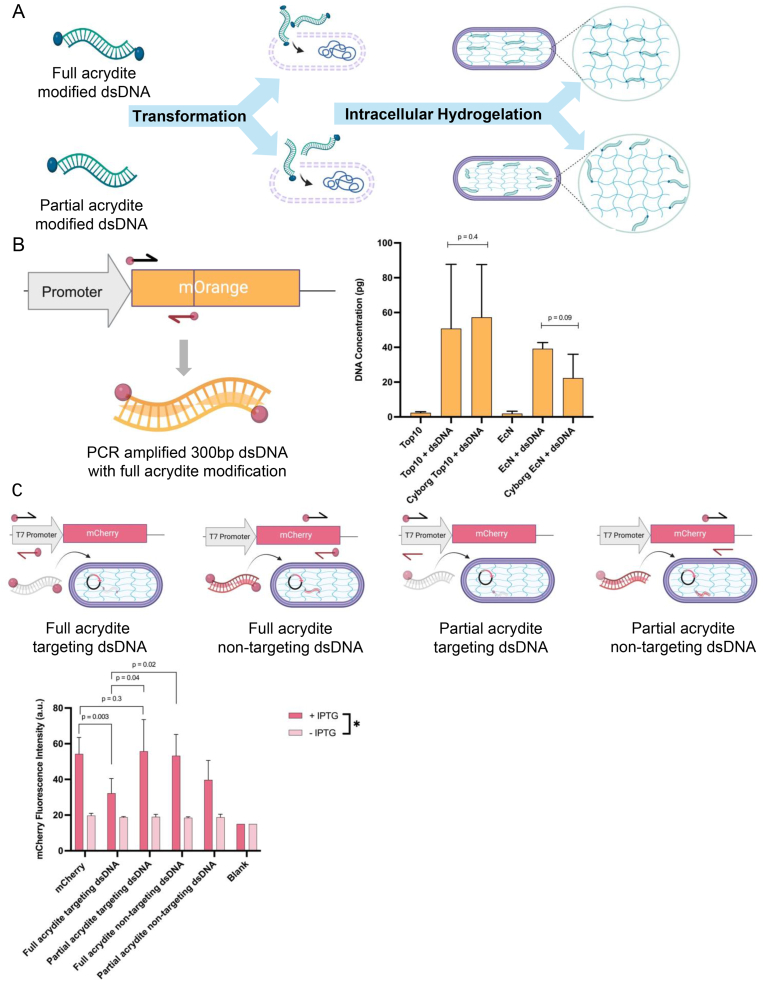
Tuning bacterial gene expression using hybrid dsDNA-PEG intracellular hydrogel. A) Schematic illustration of full and partial acrydite modified dsDNA crosslinking with hydrogel buffer. B) Concentrations of the 300-bp mOrange dsDNA inside bacteria, acquired from qPCR assay. dsDNA in the Cyborg Top10F′ and Cyborg EcN is 21.95-fold and 11.58-fold higher than negative controls Top10F′ and EcN respectively. No significant difference is observed between dsDNA transformed Top10 and Cyborg Top10, as well as, between dsDNA transformed EcN and Cyborg EcN (error bar = SD, n = 4 biological replicates. See also Fig. S5). C) mCherry expression in Cyborg and control BL21 (DE3). mCherry targeting and non-targeting partial and full acrydite-modified dsDNA are incorporated into intracellular hydrogel. Fluorescence intensity of IPTG-induced mCherry is reduced in the presence of full acrydite targeting dsDNA (error bar = SD, n = 2 biological replicates with 2 technical replicates within each biological replicate. See also Fig. S6).

Antisense oligonucleotide sequences are homologous nucleic acids that can recognize transcripts and control gene expression *in vivo* through sequestration or binding site inhibition [[40], [41], [42]]. Nucleic acid PEGylation is commonly used in extracellular polyacrylamide-based structures, but it has not been applied for intracellular mesh control [43,44]. Along this line, we investigated if intracellular PEG-DA can interface with and control cellular network dynamics. We specifically studied the effect of dsDNA-PEG gel interactions to regulate gene expression in living cells, and further incorporated this activity into functionalized intracellular hydrogels (Fig. 4A).

Because the dsDNA-PEG intracellular hydrogel is a novel intracellular complex, we first established the necessary conditions to construct, detect, and apply intracellular dsDNA-PEG hydrogels. We generated a 300bp acrydite end-modified dsDNA template through PCR amplification based on prior works [41], which consisted of a pair of primers (Sup. Table 2) flanking the coding region of an exogenous mOrange protein. Following the generation of the acrydite end-modified dsDNA, we transformed the short linear oligonucleotides into the *E. coli* cells (not containing any homologous mOrange sequences), followed by incubation with M_n_ 700 PEG-DA for intracellular hydrogelation (Methods Section M8). Initial characterization of the dsDNA-PEG hydrogel with internal Cyanine5 dye proved challenging, as concentration and steric inhibition of fluorescent probes limited the sensitivity of the system to detection. We switched to using qPCR with SYBR Green, which generated highly sensitive and accurate readouts of the target DNA sequence after cell lysis (Fig. S5A) and confirmed the accuracy of the sequence with a melting temperature analysis (Fig. S5B).

With the 300bp template dsDNA-PEG hydrogel, we determined the CT value and corresponding DNA concentration using a serial dilution of the target template ranging from 10 ng–0.01 ng (Figs. S6A and S6B). With this concentration curve, we generated Cyborg EcN and Cyborg Top10F′ and compared target DNA concentration to non-Cyborg Bacteria (with the dsDNA transformed and without mOrange plasmid template) and wildtype cells (without any dsDNA) (Methods Section M9). Top10F′ (a mcrA, mcrBC, and mrr mutant) was tested alongside EcN due to their high transformation efficiency and plasmid expression [45]. As a negative control for the dsDNA, wildtype (WT) EcN showed the lowest DNA concentration at 1.63 pg/μL DNA as compared to non-Cyborg EcN, which confirmed detectable target dsDNA with 39.1 pg/μL DNA (nearly 24-fold increase over WT EcN). The target dsDNA was also confirmed in the Cyborg EcN, with 18.5 pg/μL of DNA (approximately 11-fold increase over WT EcN). WT Top10F′ showed a similar pattern with only 2.63 pg/μL of DNA, compared to 42.9 pg/μL and 52.1 pg/μL of DNA in the non-Cyborg and Cyborg Top10F′ (Fig. 4B). Furthermore, we measured dsDNA concentration of the 300bp template dsDNA in EcN over 5 days to evaluate the stability of the DNA in Cyborg Bacteria. WT EcN control did not have the dsDNA and non-Cyborg EcN was transformed with dsDNA. After normalization to the DNA from Day-1, we observed a decreasing trend of −2.2196 pg/μL per day with a coefficient of determination at 0.8647 in the non-Cyborg EcN. Meanwhile, Cyborg EcN confirmed stable dsDNA presence with almost no change in dsDNA concentration after 5 days (Fig. S5C). With an 18-fold and 11-fold increase in Cyborg Top10F'and Cyborg EcN over WT Top10F′ and EcN respectively, we confirm that the dsDNA was integrated successfully into the Cyborg Bacteria and had high stability inside two separate Cyborg Bacterial strains.

We next established the transcriptional regulation potential of dsDNA-PEG. We amplified a 300-bp region upstream of mCherry reporter protein from pET-15b mCherry plasmid [46] containing T7 promoter, which was our “targeting-dsDNA”. We also generated a 300bp “non-targeting-dsDNA” as a scrambled control. We additionally generated two structural forms of the dsDNA templates by varying the acrydite functional groups on the primer pairs; either the forward primer only contains an acrydite modification (partial-acrydite) or both forward and reverse primers contain the acrydite modification (full-acrydite) (Fig. 4C). Previous work has shown that modulation of dsDNA-PEG meshes is possible by altering the 5′ and 3’ end of oligonucleotides with acrydite functional groups [47]. Prior works have shown that the location of integration of oligonucleotides within the dsDNA-PEG network depends on the end modifications [43]. Similarly, the full-acrydite oligonucleotides would integrate into the internal PEG mesh to directly act on and sequester native polymerases, while the partial-acrydite oligonucleotides would locate on the extreme ends of each PEG-DA polymer terminating the “elongation” of PEG-DA polymer. Based on similar oligonucleotide therapeutics, we expect that the targeting-dsDNA will sequester native T7 polymerase and reduce the overall pool of available proteins for transcription from the T7 promoter mCherry reporter, decreasing total fluorescence signal [48,49] without restricting native bacterial polymerase. We generated the dsDNA-PEG hydrogel in Cyborg *E. coli BL21 DE3* (BL21) with plasmid pET15b-mCherry, in which mCherry expression was induced by 1 mM Isopropyl ß-D-1-thiogalactopyranoside (IPTG). BL21 was utilized in this assay due to the high recombinant protein expression under IPTG inducible T7 polymerase [50]. We induced expression in Cyborg Bacteria and measured total fluorescence over 24 h, comparing the targeting and non-targeting dsDNA, as well as the partial and full acrydite-modified constructs (Methods Section M10).

The results show that WT mCherry Cyborg Bacteria (without dsDNA) display fluorescence of 54.23 a.u. (mean, n = 4) 14 h post-induction, compared to mCherry full-acrydite targeting-dsDNA Cyborg Bacteria, which displayed a significant reduction in fluorescence at only 32.25 a.u. (approximately 40 % decrease in fluorescence). Cyborg Bacteria with full-acrydite non-targeting-dsDNA (53.25 a.u.) showed no significant change in fluorescence compared to WT mCherry Cyborg Bacteria (1.8 % reduction in fluorescence). Along this line, the Cyborg Bacteria with full-acrydite targeting-dsDNA showed a significant decrease in fluorescence compared to the Cyborg Bacteria with partial-acrydite targeting-dsDNA (39.44 % reduction in fluorescence). We next confirmed a noticeable decrease in fluorescence of 44.75 a.u. in the partial-acrydite targeting-dsDNA Cyborg Bacteria compared to the WT mCherry Cyborg Bacteria, but this construct showed only an approximate 18 % reduction in fluorescence. Lastly, no significant decrease was observed in the partial-acrydite 300bp non-targeting-dsDNA compared to WT (Fig. 4C). These results indicate that we can generate a synthetic dsDNA-PEG hybrid hydrogel inside living bacteria, which maintain function according to sequence and structure/orientation. Further, the targeting dsDNA template reduces gene expression of T7 controlled mCherry reporter compared to Cyborg Bacteria lacking the target-specific and fully acrydite-modified dsDNA.

## 3 Discussion

Our work demonstrates the adaptability of the Cyborg Bacterial platform to new hydrogel compositions, utilizing an alternative photoinitiator, LAP, alongside with varied PEG structures and molecular weights. We investigate the physical and biochemical multifunctionality of a new type of biohybrid cells, creating a new frontier of modulating intracellular gel architecture to control the functions of Cyborg Bacteria. The work also demonstrates the adaptability of the biohybrid cellular platform to different synthetic materials. These aspects of Cyborg Bacteria and intracellular hydrogelation have not been explored in the literature. The choice of LAP, favored for its enhanced water solubility and rapid UV-A light-induced polymerization, results in a more efficient and potentially less toxic hydrogelation process compared to the I2959-based system. LAP, even though water soluble and less toxic, has complex synthesis procedure and is less readily accessible. Hence, we used the more accessible I2959 in some of our experiments. In our investigation, we employed RCD to detect the respiratory activity of bacterial cells as an indicator of aerobic viability. Previously, we have shown that Cyborg Bacteria maintain gene expression and inducibility from 12 different small molecules [20]. Hence, the intracellular reductive potential was sufficient to sustain a quantitative analysis and confirmation of metabolic activity in this study. However, in addition to RCD, incorporating complementary metabolic assays in further studies could enrich the characterization of the bacteria's metabolic state and provide a more comprehensive view of Cyborg Bacterial metabolic landscape.

Our study showed that the 2arm-PEG/LAP45 and 2arm-PEG/LAP65 formulations notably improved hydrogelation efficiency, as confirmed by flow cytometry, without compromising bacterial metabolic activity. The observed discrepancy between the data for fluorescein geometric mean and the percentage of fluorescein can be attributed to their fundamentally distinct concepts in our study. While fluorescein geometric mean indicates the quantity of how well bacterial cells are hydrogelated, the percentage of fluorescein provides a measure of how many cells in a population are intracellularly gelated [51]. Given that intracellular hydrogelation is not homogeneous, as seen in some TEM images (Figs. S1A and S1B), geometric mean of fluorescein might not always correlate with percent hydrogelation. Therefore, recognizing the divergence between the two metrics is essential for evaluating the extent and uniformity of hydrogelation within individual cells, informing the optimization of the Cyborg Bacterial platform for its intended applications.

The integration of 4arm-PEG Acrylate M_n_ 1000 into the Cyborg Bacterial platform confirms its capacity to incorporate different molecular architectures while preserving bacterial functionality. The branched structure of 4arm-PEG Acrylate M_n_ 1000 was hypothesized to facilitate denser hydrogel networks. This hypothesis was supported by our results, as the 4arm-PEG hydrogels showed a significant increase in the metabolic activity of Cyborg Bacteria compared to 2arm-PEG hydrogels (Fig. S3B). This result agrees with previously published work, identifying 4arm-PEGDA hydrogels by their characteristic dense network and large pore sizes (the branched structure creates more open spaces within the hydrogel network), which can aid in molecule trafficking and cell function. In addition, decreasing PEG molecular weight shows a decrease in Cyborg Bacteria activity. This result is consistent with previously published articles, which show a decrease in hydrogel pore size [31] that can further restrict molecular diffusion. These results established M_n_ 700 2arm-PEG as the lower limit and M_n_ 1000 4arm-PEG as the upper limit for optimal intracellular hydrogelation, ensuring cell vitality and functionality.

Lastly, we explored the potential of PEGylated oligonucleotide sequences in intracellular hydrogels to regulate gene expression within living cells. While previous work has focused on intracellular DNA-PEG conjugates to drive or enhance gene regulation, our work is the first to utilize fully mesh-integrated oligonucleotide-PEG hybrid hydrogel. Our goal in this study was to observe and characterize the phenotypic profile of Cyborg Bacteria with variable structural and sequence-specific intracellular dsDNA-PEG hydrogels, which we confirmed through post-translational control of gene expression. Our modified dsDNA-PEG hydrogel system integrated effectively within cells, as confirmed by qPCR. Further studies could explore the structural and physical properties of the purified dsDNA-gel from Cyborg Bacteria. However, this research will require advanced changes of current X-ray crystallography or intracellular enhanced Raman spectroscopy to delineate intracellular dsDNA-PEG structures and enhance the signal-to-noise ratio. Our modified dsDNA-PEG hydrogel also modulated gene expression, reducing mCherry fluorescence with full-acrydite targeting dsDNA structures. Double-stranded DNA with full acrydite modification was explored in previous literature, indicating no significant loss in enzymatic access/activity to the DNA strands over time, and stability of the hydrogel could persist longer in the presence of PEG-DA at multiple linear or branching sites on either end [52,53]. This suggests that dsDNA-PEG hydrogels could enhance the long-term stability of internalized DNA, granting a protective effect while maintaining therapeutic activity. These observations underscore the importance of complete DNA integration within the structural matrix for effective gene sequestration and demonstrate a potential for targeted gene regulation within this novel intracellular matrix. In future work, researchers can potentially modify the specific nucleotide sequence integrated into the Cyborg Bacteria to sequester certain transcription factors. This assumption is based on prior works that modify various RNA and oligonucleotides for targeted gene silencing, including end-modification of miRNA to enhance suppression of genes [54,55].

Altogether, our experiments demonstrate the adaptability of the Cyborg Bacterial platform to various hydrogel compositions and molecular architectures that could enable further modification of Cyborg Bacteria for the delivery of therapeutics and microbiome applications. Cyborg Bacteria are a promising biomedical platform as they combine the benefits of living cells with the advantages of synthetic materials. Traditional methods in cell therapy, tissue engineering, and synthetic biology typically rely on genetic and chemical modifications to enhance biocontainment. However, these methods can face challenges in control and reproducibility. By integrating synthetic polymers into cellular structures, Cyborg Bacteria offer new opportunities in cellular engineering, including modulating cellular states, restricting replication, and creating unique biomimetics [56]. Through material-cell interfaces, we can alter and enhance the adaptability of live cells with synthetic components, enabling precise control over cellular behavior and fate. Alternatively, complex and semi-functional cell-material interfaces explore the potential of cell-mimicking biomaterials. By enhancing intracellular materials with live cells, we can generate programmable biomaterials capable of performing tasks, such as drug delivery, without the risks associated with live cell use, such as uncontrollable replication or mutation. This work represents a new benchmark for intracellular hydrogel systems that can be fine-tuned for desired applications. Furthermore, our platform accommodates a balance between structural integrity and cellular health, offering enhanced control over cellular states that surpasses the limited functionality and stability of artificial cells in diverse environments. This work paves the way for the design of advanced biomaterials tailored to enhance cellular function and survival, which has implications for tissue engineering, drug delivery, and synthetic biology.

## 4 Methods

### 4.1 M1: Construction of plasmids and strains

*Strains: Escherichia coli* strains and plasmids used in this study are summarized in the table (See Sup. Table 1). *E. coli Top-10* cells (ThermoFisher) were used primarily for plasmid cloning and maintenance, as well as a diverse host for dsDNA-PEG hydrogel formation. We used the strains *E. coli BL21* (DE3), *E. coli Nissle 1917*, (*E. coli* ‘Migula’ Castellani and Chalmers (ATCC 25922™)) as the main strains for reporter expression and Cyborg Bacterial formation. All cells were cultured overnight in 2x YT media at 37 °C shaking at 250rpm. We created the *E. coli BL21 DE3* pET15b-mCherry by chemically transforming the plasmid pET15b-mCherry into the cells and selecting with Carbenicillin (100 μg/mL). The transformed cells were then prepared for secondary transformation and hydrogelation to create the Cyborg dsDNA-PEG cells.

*Plasmids:* In this study, the fluorescent reporter plasmid (pET15b-mCherry) was ordered from Addgene alongside the template plasmid for non-coding/exogenous dsDNA from pSC101-mOrange.

### 4.2 M2: Construction of Cyborg Bacterial Cells

*E. coli* Nissle was hydrogelated using the same core protocol with modifications to account for photoinitiator and PEG variants. 2arm/I2959 hydrogel buffer was prepared by mixing together 800 μL poly(ethylene glycol) diacrylate (PEG-DA; M_n_ = 700 Da; Sigma-Aldrich), 160 μL 2-hydroxy-4′- (2-hydroxyethoxy)−2-methylpropiophenone (0.75 g/u in DMSO; I2959; Sigma-Aldrich) photoinitiator solution, and 28 μL fluorescein O,O′-diacrylate (36 mg/mL in DMSO; Sigma-Aldrich). 2arm/LAP85,65,45 hydrogel buffers were prepared by mixing 358 μL, 267 μL and 176.5 μL Lithium phenyl-2,4,6-trimethylbenzoylphosphinate (LAP; 85,65,45 mg/mL in water; Millipore Sigma) with 614 μL, 704 μL and 795 μL PEG-DA respectively to final concentrations of 93.14, 53.15 and 24.32 mM LAP in the final buffer mixture. 28 μL fluorescein O,O′-diacrylate was added in each of the three buffers. 4arm PEG1K/I2959 hydrogel buffer was prepared by mixing 80 μL of 4arm PEG1K (custom synthesized; JenKem Technology) with 720 μL of DMSO, 160 μL of I2959 and 28 μL of fluorescein O,O′-diacrylate. 4arm PEG1K/LAP65 hydrogel buffer was prepared by mixing 70.4 μL of 4arm PEG1K with 633.6 μL DMSO, 267 μL LAP65 and 28 μL fluorescein O,O′-diacrylate. All the different component tuned hydrogel buffers were vortexed for 20 min to achieve a homogeneous mixture.

EcN was grown overnight in 3 mL of 2x YT media at 37 °C with shaking at 250 rpm and supplemented, if necessary, with kanamycin 30 μg/mL (Only for *E. coli* BL21 (DE3) transformed with the plasmid pET15b mCherry). The overnight cultures were diluted 5-fold using 2x YT media with the appropriate antibiotics and incubated for ∼1 h until the culture reached an OD_600_ of 0.6-0.8. After reaching the appropriate OD_600_, cells were spun down (4000g, 10 min, 20 °C), resuspended in 2x YT media without antibiotics at a cell density of 0.2 g/mL, aliquoted into 1.5 mL microcentrifuge tubes (ThermoFisher Scientific), and incubated with hydrogelation buffer (1 WT% LAP or I2959 photoinitiator and 5 % any PEG variant) for 30 min at 37 °C with constant rotation at 0.125 Hz on a rotary axis such that the tubes were inverted with each rotation. After incubation with the hydrogel buffer, bacterial cells were flash frozen by submerging the vials in supercooled methanol at −80 °C for 2 min. Cells were then incubated at −80 °C for 10 min, and then thawed at 30 °C in a dry bath for about 5 min. Vials with bacterial cells were immediately spined down after thawing (6800g, 10 min, 20 °C), and washed twice using fresh 2x YT media without antibiotics. Bacterial cells infused with hydrogel were then crosslinked with UV light using an energy delivery of 1600 mW/cm^2^ (6 min 3 s) for I2959 based hydrogels and 800 mW/cm^2^ (2 min 58 s) for LAP based hydrogels (Light source: UVP Crosslinker CL-3000L - Longwave (365 nm), 115V, Analytik Jena GmbH). Following UV irradiation, cells were spun down (6800g, 10 min, 20 °C), and washed twice using 1X PBS buffer. After the final wash, Cyborg Bacteria were resuspended in an appropriate media for further experiments. WT EcN cells, as a negative control in all experiments, despite not containing hydrogel buffer, were subjected to the hydrogelation protocol, which includes the freeze/thaw and UV exposure steps.

### 4.3 M3: Optimization of the LAP based hydrogels’ polymerization rate under varying UV intensities

LAP was resuspended in quantities of 45, 55, 65, 75, and 85 mg in 1 mL of deionized water. After mixing the solutions, they were combined with M_n_ 700 PEG-DA. Both the buffer alone and in combination with 2x YT media (67 μL buffer and 933 μL 2x YT media), to mimic the cell encapsulation conditions, were subjected to six distinct UV intensities (1600, 1000, 800, 600, 400, and 200 mW/cm^2^) inside the UVP crosslinker. The crosslinked gels were visually inspected for their breakability to assess the most optimum UV intensity. 800 mW/cm^2^ was selected as the optimum energy to crosslink LAP-based hydrogels (Fig. S2E).

### 4.4 M4: Flow cytometry

Cyborg EcN cells were hydrogelated, and pellets were resuspended in 1X PBS. OD_600_ of the WT and Cyborg Bacteria were normalized to 0.1. Samples were stained for reductive chromogenic dye by adding 20 μL CTC and 1 μL enhancing reagent A (Dojindo Laboratory) components of the kit into 1 mL of 0.1 OD_600_ bacterial samples. Cells were incubated at 37 °C for 1 h on a rotating rack. As a negative control for metabolic activity, OD_600_ 0.1 WT EcN cell pellet was fixed using 1 mL of 4 % PFA (vol/vol, ThermoFisher Scientific) for 20 min at room temperature, followed by twice washes with 1X PBS and RCD staining. Samples were analyzed on Beckman CytoFLEX S flow cytometer with 488 nm blue laser excitation coupled with 525/40 nm (for measuring fluorescein-PEGDA) and 695/40 nm bandpass filters (for measuring RCD). Data analysis was performed with the Flowjo software, with geometric means calculated based on the log-distributed datasets. Fluorescent Cyborg EcN were gated based on non-fluorescent WT EcN controls and on laser light scatter (forward vs. side angle scatter) to exclude debris and aggregates.

### 4.5 M5: CFU and FACS sorting

The proliferation capabilities of the resulting WT and Cyborg EcN cell populations were assessed by CFU assays before and after fluorescence activated cell sorting (FACS). To sort Cyborg Bacterial cells, the OD_600_ of WT and Cyborg EcN were normalized to 0.05 in 1 mL 1X PBS. Single, fluorescein positive bacteria were selected and sorted by the Beckman Coulter “Asterios” cells sorter assisted by technical personnel at the sorting facility. The cell sorter was pre-configured for rapid processing of the bacterial populations using the 70 μm nozzle at 60 psi fluid pressure. Non-fluorescent and fluorescent bacteria were sorted by 488 nm laser and gated on laser light scatter (forward vs. side angle scatter) to exclude debris and aggregates. The putative, single non-fluorescent bacterial controls were assessed using the 488 nm laser to set background levels of autofluorescence measured into a 529/30-restricted photodetector prior to analysis of test samples at identical sensitivity settings. A total of 1.5 × 10^6^ fluorescein-PEGDA positive Cyborg and WT EcN cells were collected for CFU analysis. Non-sorted Cyborg EcN cell density was normalized to 1.5 × 10^6^ cells and CFU dilution plates were prepared. Briefly, the resulting sorted and non-sorted 1.5 × 10^6^ cells were diluted 10^−1^ -10^−8^ times and 10 μL were plated in duplicates into individual spots on a petri dish (Sigma-Aldrich) with 2x YT agar. Plates were incubated overnight (37 °C) and the resulting colonies in the appropriate dilutions were counted and reported as CFUs/mL accounting for the dilution factor and the volume used to plate. For accuracy and consistency, we only counted colonies in a dilution if there were between 5 and 50 colonies. A result of less than 5 colonies was considered as below the detection level.

### 4.6 M6: Fluorescence microscopy

WT and Cyborg EcN samples were imaged using lab-made 1.5 % Low Melting Temperature Agar (LMTA), 1X PBS gel pads (50 × 25 mm, Thickness: 1 mm). Gel pads were mounted over glass slides (Plain Micro Slides, 75 × 50 mm, Thickness 1 mm, Corning Inc) and divided into 2 individual squares to allow for imaging of 2 separate samples at once. 10 μL OD_600_ of 0.1 normalized WT and Cyborg EcN samples were pipetted into the center of each square and a cover slip was placed over the gel pad. Fluorescence microscopy images were acquired using a Nikon Eclipse Ti inverted fluorescence microscope with perfect focus 3 (Nikon Instruments Inc) equipped with a 100x/1.4 oil objective. Exposure times were fluorescein: 300 ms, and Bright Field: 56 ms. Microscope filter settings were fluorescein, excitation, 450–490 nm; emission, 500–550 nm; gain ≥495 nm. Images were analyzed with open-source platform for biological imaging analysis Fiji (http://fiji.sc/cgi-bin/gitweb.cgi/).

### 4.7 M7: Sample preparation for TEM microscopy and analysis

The pellets of WT and Cyborg EcN cells were fixed in a fixative solution (2.5 % glutaraldehyde, 2 % paraformaldehyde in 0.1 M sodium phosphate buffer). After fixation, samples were rinsed twice in 0.1 M sodium phosphate buffer for 30 min and placed in 1 % osmium tetroxide in 0.1 M sodium phosphate buffer for 1 h. Samples were rinsed 2 times for 15 min each in 0.1 M sodium phosphate buffer. Samples were then dehydrated in 50 % EtOH, 75 % EtOH, 95 % EtOH (at least 30 min each), and finally in 100 % EtOH (2 × 20 min). Samples were placed in propylene oxide 2 × 15 min and were pre-infiltrated in half resin/half propylene oxide overnight. On the next day, samples were infiltrated in 100 % resin (composed of 450 mL dodecenylsuccinic anhydride, 250 mL araldite 6005, 82.5 mL Epon 812, 12.5 mL dibutyl phthalate, and 450 μL benzyldimethylamine) for 5 h. The samples were embedded with fresh resin and polymerized at 65 °C overnight. The embedded cells were sectioned with a Leica EM UC6 ultramicrotome at a thickness of 90 nm and collected on copper mesh grids. The sections were stained with 4 % aqueous uranyl acetate for 20 min and for 2 min in 0.2 % Lead Citrate in 0.1 N NaOH. TEM imaging was done on FEI Talos L120C at 80kv using ThermoFisher Scientific Ceta 16 MP camera. Images were analyzed with Fiji ImageJ, calculating mean pixel intensity per cell in each 11000x frame for all cell conditions.

### 4.8 M8: dsDNA-PEG hydrogelation

*dsDNA Template:* Four sequences were explored and amplified to prepare the dsDNA-PEG hydrogel through acrydite-modified oligonucleotide templates (IDT). For sequestration/homology binding, T7 promoter sequences, and overlapping ORF were amplified using OneTaq 2x Master Mix (NEB). pET15b-mCherry (Addgene) possesses a T7-inducible promoter, and approximately 100bp upstream of the RBS, the forward primers ‘T7 F’ and ‘T7 F-AC’ amplifies the T7 promoter and 5′UTR, extending 300bp into the ORF for the corresponding reverse primer pair ‘T7 R’ and ‘T7 R-AC’ for non-acrydite-modified and acrydite-modified dsDNA. Non-targeting/scrambled DNA elements were amplified from the terminator sequence, extending 300bp into the ORF using primers ‘NT F’ and ‘NT R’ for non-acrydite-modified dsDNA and ‘NT F-AC’ and NT R-AC’ for acrydite-modified dsDNA. Additionally, the non-homologous 300bp mOrange template was amplified from plasmid pSC101-mOrange (Addgene), with sequences sharing no continuous DNA homology to the coding dsDNA template greater than 10bp.

The process began with an overnight culture growth at 37 °C in 20 mL of 2x YT media. Subsequently, the cells were pelleted, made chemically competent using a Zymo Mix & Go *E. coli* Transformation kit, and transformed with prepared dsDNA at a concentration of 500 ng per 5 × 10^6^ cells per mL. A 5 % w/v 700 M_n_ PEG-DA buffer served as the gel basis and crosslinked using UV light (1600 mW/cm^2^) and 2-hydroxyl-4′-(2-hydroxyethoxy)-2-methylpropiophenone to minimize biological interaction. For the purposes of excluding fluorescent signal, we forewent the addition of 0.05 % w/v fluorescein O’O – diacrylate in the buffer, as this would interfere with the identification of target dsDNA via SYBR Green PCR Amplification.

### 4.9 M9: qPCR detection

The presence of the dsDNA-PEG hydrogel was confirmed through gel electrophoresis and qPCR. Small targeted sequences were isolated via OneTaq 2x MasterMix PCR amplification (NEB) using coding sequence paired primers ‘T7 F’ and ‘T7 R’ and ‘NT F’ and ‘NT R’ (without end modifications) with a 50 °C annealing temperature and 20 s extension time for 35 cycles. The resulting fragments were purified using a PCR Cleanup Kit (NEB) and run through a 1 % agarose gel at 100V for 45 min to identify target bands at 300bp length (data not shown). SYBR Green amplification in a ViiA7 qPCR machine was utilized to quantify the target dsDNA, estimating nucleic acid concentration per 4 μL of cell lysate to gauge transformation efficiency and gelation (Sup. Table 3). Negative controls were prepared by transforming linear mOrange dsDNA into bacteria not containing any plasmid or mOrange homology to potentially amplify and produce false positives. Results from melting temperature were compared to approximated T_m_ calculated from Benchling, and amplification results were analyzed via Quantstudio Design and Analysis 2.7.0 (Fig. S5B). A template DNA amplification was diluted from 10 ng including 1 ng, 0.1 ng, 0.01 ng, and 0.001 ng to generate a standard curve to evaluate approximate DNA concentration per CT value (Figs. S6A, S6B, S6C). The stability of full-acrydite mOrange dsDNA in EcN was similarly tracked over 5 days, storing the freshly transformed and hydrogelated bacteria in PBS at 4 °C, lysing at 98 °C for 6 min, and purifying 4 μL of cell lysate for qPCR after 1 day, 3 days and 5 days.

### 4.10 M10: Plate-based fluorescence screening

Cyborg *BL21-*mCherry with the corresponding dsDNA-PEG hydrogel constructs, were incubated in 1 mM IPTG in 200 μL of 2x YT growth media with 50 mg/mL D-Cycloserine in a 96-well black clear-bottom plate (Corning) to observe mCherry fluorescence at 587 nm excitation, 610 nm emission and gain of 100. The fluorescence intensity was monitored every 10 min for 18 h at 37 °C, with orbital shaking at an amplitude of 3 mm for 1 min using an m1000Pro Infinite plate reader (Tecan). Successful gene downregulation, indicated by cells expressing mCherry fluorescence, was determined by the sequestration of T7 RNA Polymerase by the designed hydrogel. To ensure accurate fluorescent signal observation, multiple controls, including Cyborg controls and non-Cyborg controls, were incorporated to account for potential autofluorescence and background, alongside Targeting versus non-Targeting dsDNA transformation without hydrogel formation and with and without 1 mM IPTG induction. Triplicates for *E. coli BL21* (DE3) fluorescence include mCherry (no inhibition), mCherry + Full-Acrydite Targeting dsDNA-PEG hydrogel ( ± IPTG), mCherry + Partial-Acrydite Non-Targeting dsDNA-PEG hydrogel ( ± IPTG), mCherry Full-Acrydite Non-targeting dsDNA-PEG hydrogel ( ± IPTG) and mCherry Partial-Acrydite Non-Targeting dsDNA-PEG hydrogel ( ± IPTG).

### 4.11 M11: ATP luminescence assay

Cyborg EcN was created by permeabilizing a hydrogel buffer composed of 2armPEG-DA and I2959 photoinitiator into bacterial cells with freeze and thaw cycle. After crosslinking the hydrogel inside the bacterial cells with UV light (1600 mW/cm^2^), WT and Cyborg EcN (OD_600_ of 0.2) were treated with 400 μg/mL carbenicillin for 2.5 h to get rid of the growing bacterial cells (carbenicillin kills growing bacterial cells, while leaving Cyborg Bacteria intact). As negative controls, heat-killed WT EcN cells (OD_600_ of 0.2, incubated at 95 °C for 30 min), PFA-treated WT EcN cells (OD_600_ of 0.2, treated with 4 % PFA for 30 min), and lysed WT EcN cells (OD_600_ of 0.2, treated with 1 % Triton X-100 for 30 min) were used. Post 2.5 h of incubation with carbenicillin, 100 μl of 10^8^ WT EcN, carbenicillin treated WT EcN, carbenicillin sorted Cyborg EcN, heat-killed EcN, PFA treated EcN and lysed EcN were mixed with 100 μl BacTiter-Glo^TM^ reagent (Promega) in Nunc 96-well optical polystyrene polymer bottom white plate (ThermoFisher Scientific) and the plate was incubated for 5 min on an orbital shaker to mix the reagent with the sample. ATP luminescence was then recorded on an m1000Pro Infinite plate reader (Tecan).

### 4.12 M12: Statistical analysis of results

Standard two-sample *t*-test, assuming unequal variances, were conducted for statistical analysis. Results were considered significant if the p-values were less than 0.05, and the p-values were incorporated in each graph, showing the comparison between individual groups. The figure legends include the number of replicates used in the calculations.

## Funding sources

1- NCI P30 CA093373 (Comprehensive Cancer Center)
2- S10 OD018223 – Beckman Coulter “Astrios” cell sorter and “Cytoflex” cytometer.
3- NIH/NIGMS R35GM142788

## CRediT authorship contribution statement

**Ofelya Baghdasaryan:** Writing – review & editing, Writing – original draft, Methodology, Investigation, Formal analysis, Data curation, Conceptualization. **Jared Lee-Kin:** Writing – review & editing, Writing – original draft, Methodology, Investigation, Formal analysis, Data curation, Conceptualization. **Cheemeng Tan:** Writing – review & editing, Writing – original draft, Supervision, Resources, Project administration, Investigation, Funding acquisition, Conceptualization.

## Declaration of competing interest

The authors declare the following financial interests/personal relationships which may be considered as potential competing interests:

Cheemeng Tan reports financial support was provided by University of California Davis. Cheemeng Tan has patent pending to Assignee. If there are other authors, they declare that they have no known competing financial interests or personal relationships that could have appeared to influence the work reported in this paper.

## Data Availability

Data will be made available on request.
