# Supplementary material for: Architectural engineering of Cyborg Bacteria with intracellular hydrogel
Source: Mater Today Bio. 2024 Sep 6;28:101226. doi: 10.1016/j.mtbio.2024.101226 (PMC11426140; doi:10.1016/j.mtbio.2024.101226)
Supplement: Multimedia component 1 [file mmc1.pdf]

Architectural Engineering of Cyborg Bacteria with Intracellular Hydrogel

Ofelya Baghdasaryan[1*], Jared Lee-Kin[1*], Cheemeng Tan[1,#]

[1]Biomedical Engineering, University of California Davis

#Corresponding authors (cmtan@ucdavis.edu)

*Equal contribution

Supplementary Figures

Fig. S1A

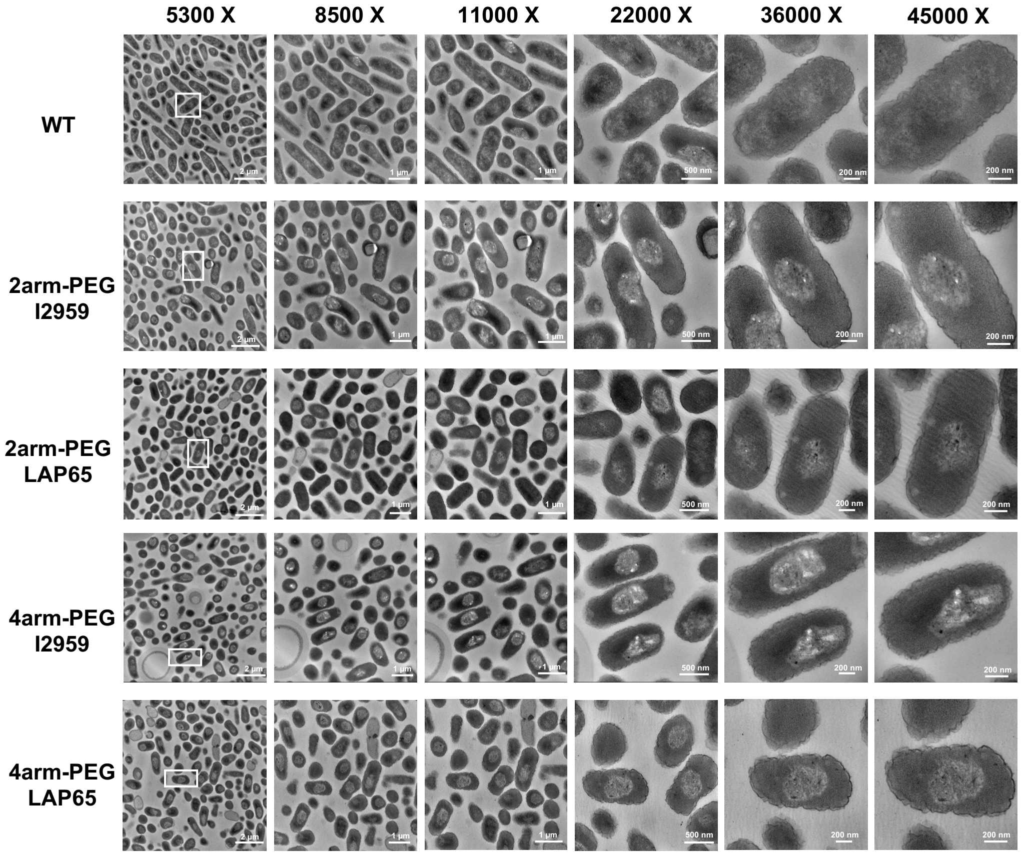

Fig. S1B

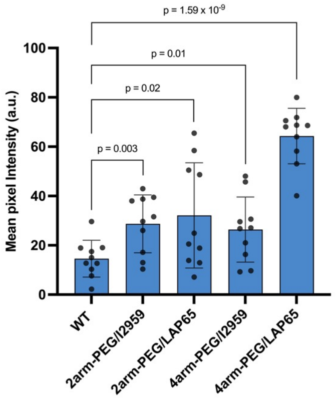

Fig S1: TEM images of Cyborg EcN cells under different hydrogel compositions.

A) From top to bottom, TEM images of WT, 2arm-PEG I2959, 2arm-PEG LAP65, 4arm-PEG I2959, and 4arm-PEG LAP65 EcN are represented at 5,300 X, 8,500 X, 11,000 X, 22,000 X,

36,000 X and 45,000 X with zooming into a single bacterium. Cyborg EcN cells exhibit denser contrasted region in the cytoplasm, as compared with WT EcN cells, indicating that the hydrogel infusion inside bacteria resulted in changes in inner cell structures (scale bar = 2 µm, 1 µm, 1 µm, 500 nm, 200 nm and 200 nm from left to right, n = 1 biological replicate).

B) Calculation of pixel intensity in WT and Cyborg EcN from TEM images (Figure S1A). Results show a higher contrast and greater pixel intensity after thresholding in Cyborg EcN with mean values of 28.7 (2arm-PEG/I2959), 32.1 (2arm-PEG/LAP65), 26.4 (4arm-PEG/I2959) and 64.3 (4arm-PEG/LAP65) compared to WT EcN with a mean value of 14.6 (error bar = SD, n = 1 biological replicate, 10 images per sample were analyzed).

Fig. S2A

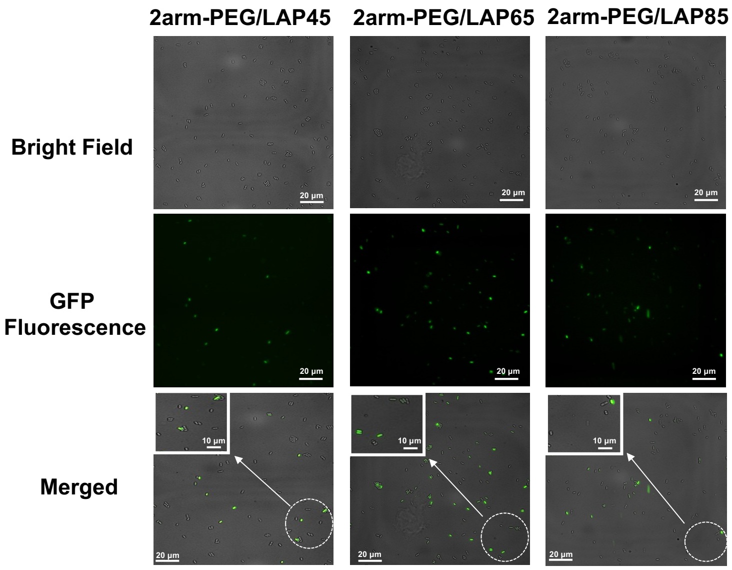

Fig. S2B

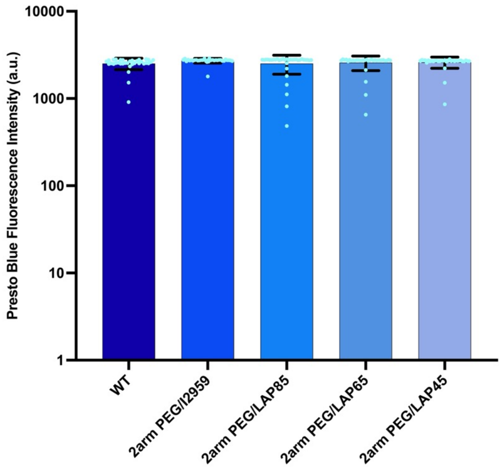

Fig. S2C

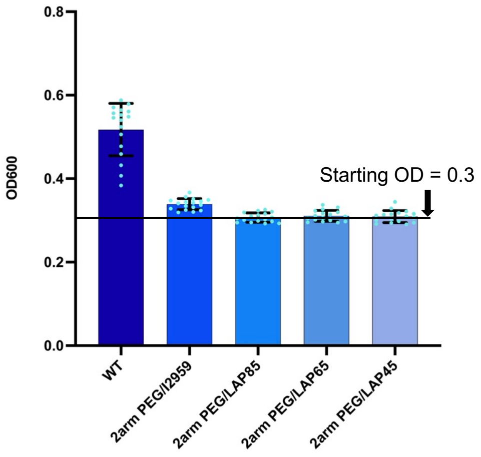

Fig. S2D

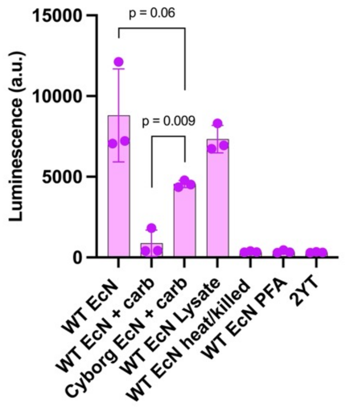

Fig. S2E

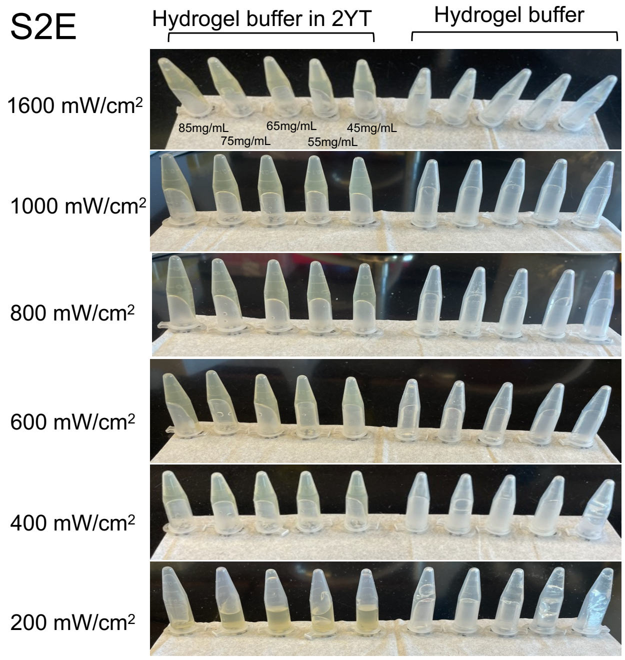

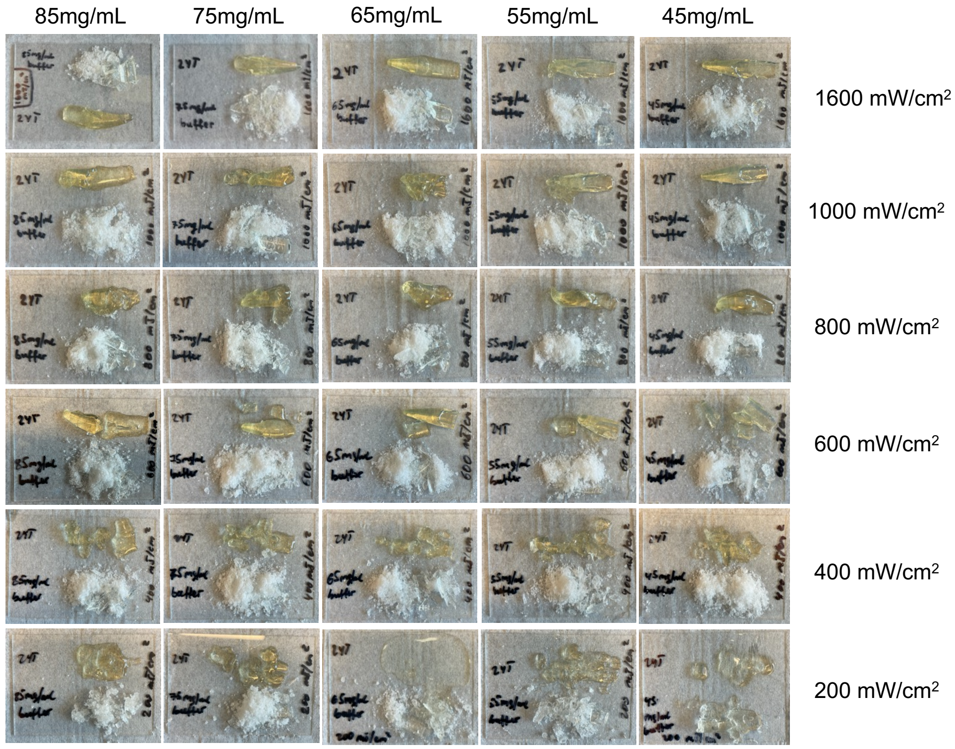

Fig S2: Characterization of 2arm-PEG hydrogels with LAP and I2959 photoinitiators.

A) Fluorescence microscopy images of Cyborg Bacteria created using 2arm-PEG/LAP45, 2arm-PEG/LAP65 and 2arm-PEG/LAP85. Images show conserved bacterial cell morphology and

fluorescence of intracellular hydrogel (Scale bar = 20 μm and 10 μm for zoomed in image, n = 3 biological replicates).

B) Mean OD_{600} of WT, as well as 2arm-PEG/LAP45, 2arm-PEG/LAP65, 2arm-PEG/LAP85 modified EcN cells. Cyborg EcN shows halted replication when compared to WT EcN cells (starting initial OD_{600} of 0.3 across all the samples, overnight measurement, error bar = SD, n = 1 biological replicate).

C) Mean metabolic activity of WT, as well as 2arm-PEG/LAP45, 2arm-PEG/LAP65, 2arm-PEG/LAP85 modified EcN. Results show sustained metabolic activity in Cyborg EcN Cells, similar to that of WT EcN cells (starting initial OD_{600} of 0.3 across all the samples, overnight measurement, error bar = SD, n = 1 biological replicate).

D) ATP luminescence assay of WT and Cyborg EcN. Carbenicillin-sorted Cyborg EcN cells show ATP levels comparable to that of WT EcN, while carbenicillin-treated EcN cells show significantly reduced ATP levels than that of Cyborg Bacteria. Heat/killed and PFA-treated EcN cells do not show detectable levels of ATP. Lysed WT EcN cells have ATP levels comparable to that of WT EcN, indicating that the ATP kit effectively measures ATP through the cell lysis process (error bar = SD, n = 3 biological replicates).

E) Optimization of the UV energy setting for hydrogel buffers made with different concentrations of LAP photoinitiator.

Top panel: From top to bottom, 6 different UV energies were applied to hydrogel buffers made with 85 mg/mL, 75 mg/mL, 65 mg/mL, 55 mg/mL and 45 mg/mL LAP (in the same order on each image panel) tested both in combination with 2YT media (right 5 tubes, 933 μL 2YT + 67 μL hydrogel buffer) to mimic the cell encapsulation conditions, as well as the hydrogel buffer alone (left 5 tubes). Post UV crosslinking at each energy, the tubes were inverted and imaged to show the formation of the buffer. At 200 mW/cm^2, no hydrogel buffer was formed in 2x YT media.

Bottom panel: Post UV crosslinking, hydrogels from each tube were removed and placed on a glass slide to visually inspect their strength and breakability, particularly in 2x YT media. Starting from crosslinking energy of 600 mW/cm^2, the hydrogel in 2x YT media was more prone

to breaking when it was being removed from the tube and contained more liquid content, while at 200 mW/cm^2, hydrogel did not fully form. At all UV crosslinking conditions, the hydrogel buffer alone was very stiff and almost impossible to remove from the tube. Hence, it was broken into pieces to place on the glass slide. 800 mW/cm^2 UV crosslinking energy was chosen as the lowest UV energy that can generate non-breakable hydrogel buffers in combination with 2x YT media.

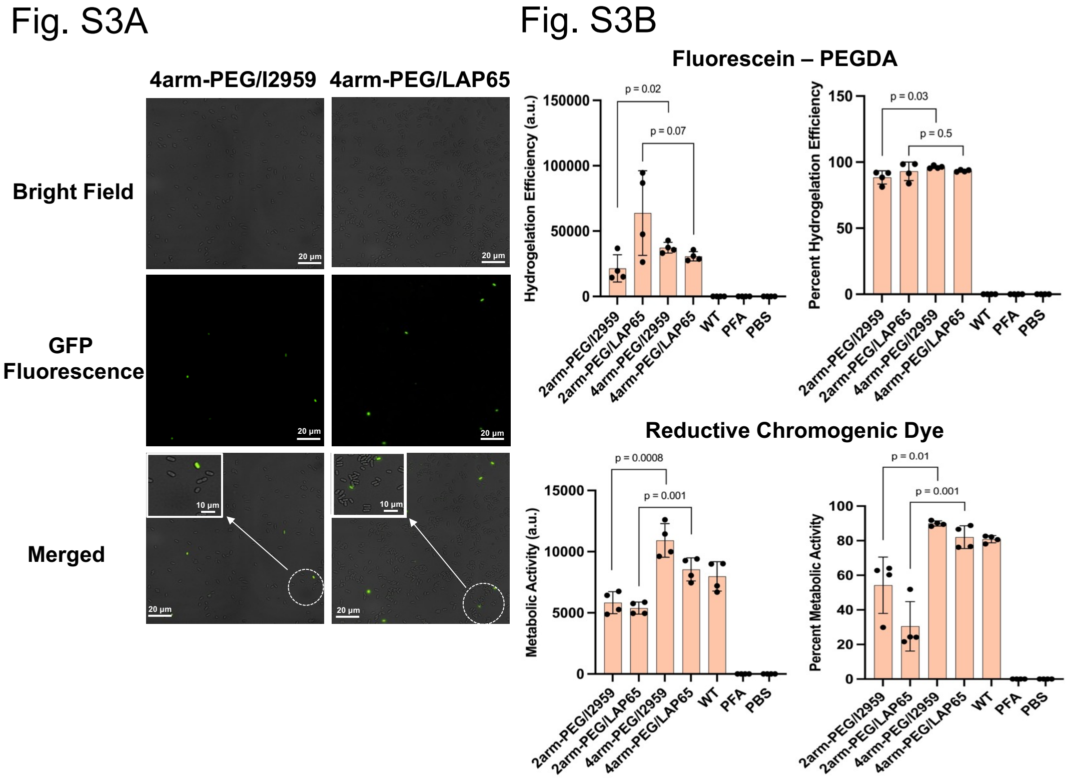

Fig S3: Characterization of 4arm-PEG hydrogels with LAP and I2959 photoinitiators.

A) Fluorescence microscopy images of 4arm-PEG/I2959 and 4arm-PEG/LAP65 show conserved bacterial cell morphology and Cyborg Bacterial phenotype (Scale bar = 20 µm and 10 µm for zoomed in image, n = 3 biological replicates).

B) Comparison between percentage and mean fluorescein-PEGDA (top panels) and RCD (bottom panels) of Cyborg EcN with 2arm-PEG and 4-arm-PEG hydrogel variants. 4arm-PEG/I2959 tuned Cyborg EcN show approximately 1.5-fold higher mean hydrogelation

efficiency than that of 2arm-PEG/I2959 Cyborg EcN. Cyborg EcN tuned with 4arm-PEG variants (I2959 and LAP65) exhibit an approximately 2-fold and 1.5-fold increase in RCD geometric mean compared to 2arm-PEG/I2959 and 2arm-PEG/LAP65, respectively.

Fig. S4

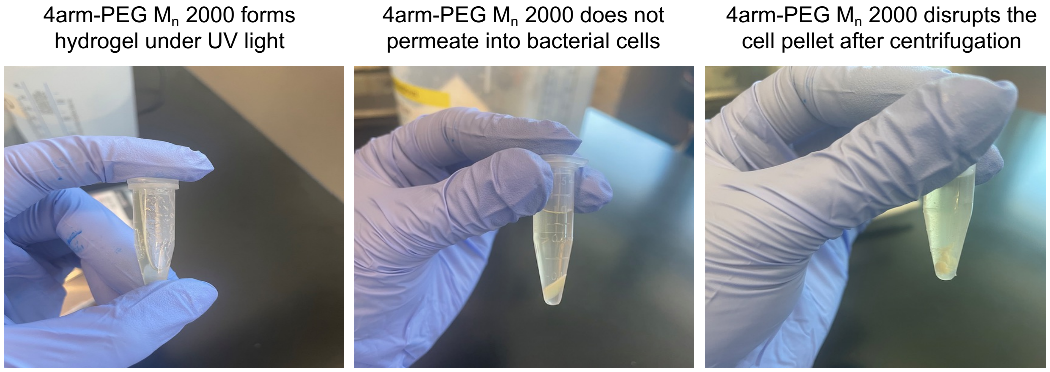

Fig S4: Intracellular hydrogelation with 4arm-PEG M_n 2000.

4arm PEG-M_n 2000 forms hydrogel under UV light (left image). It does not permeate into EcN cells after the freeze-thaw cycle, evidenced by a thick coating of the hydrogel on the surface of the bacterial cells and at the bottom of the tube (middle image). 4arm-PEG M_n 2000 disrupts the bacterial pellet after centrifugation, as evident from the cell debris formed at the bottom of the tube (right image).

Fig. S5A

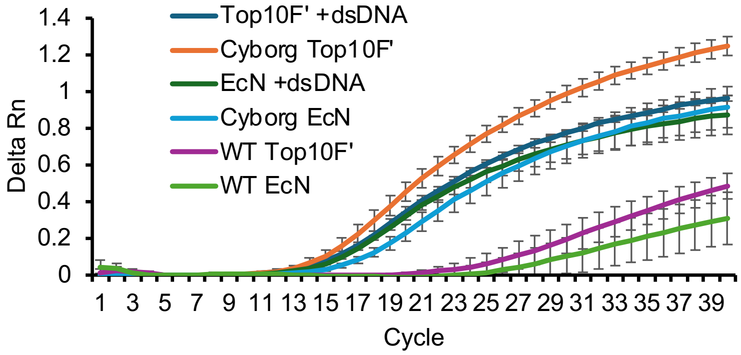

Fig. S5B

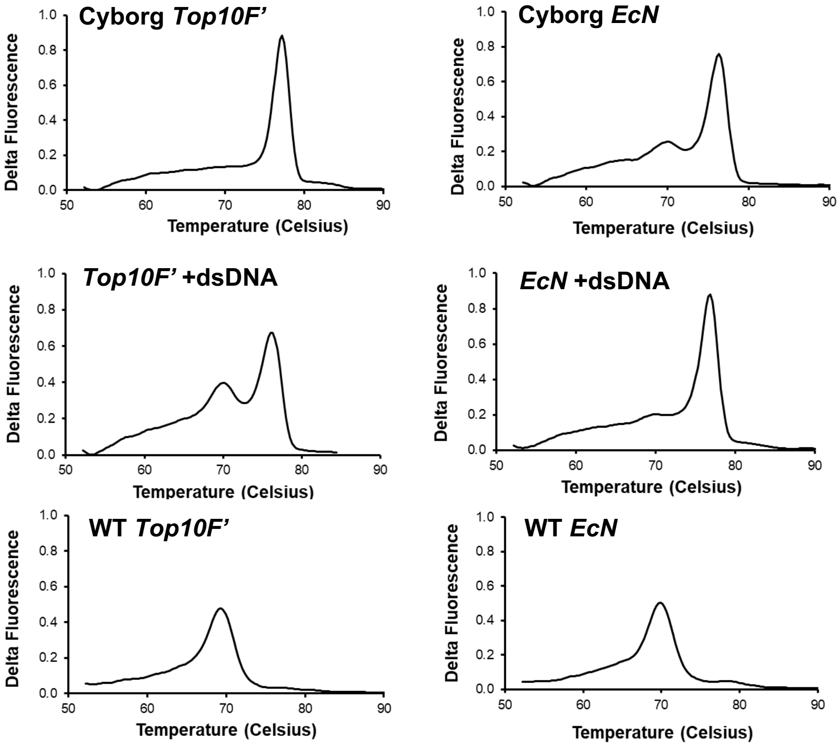

Fig. S5C

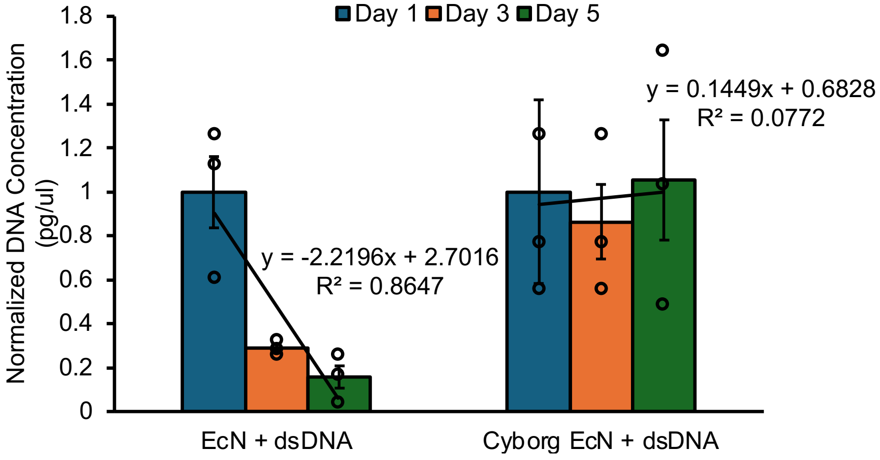

Fig S5: qPCR identifies the synthetic dsDNA-oligo incorporated inside Cyborg Bacteria

A) qPCR amplification curves of 300bp mOrange dsDNA in Top10F' and EcN. The dsDNA oligo was transformed into bacteria and is present in both Cyborg and non-Cyborg populations compared to WT EcN and Top10F'. The results show amplification of dsDNA mOrange in cells containing the dsDNA oligo (error bars = SD, n = 3 technical replicates).

B) Melting curves of amplified mOrange dsDNA oligonucleotides (76.5°C Melting Temperature) from the qPCR. The results show the corresponding melting temperature in the samples with the target mOrange dsDNA oligo.

C) dsDNA-PEG stability assay of 300bp mOrange dsDNA in EcN cells. Incubated in 1X PBS at 4°C over 5 days. The measured concentration of target DNA on Day 1, Day 3, and Day 5. The results show a clear decreasing trend of DNA concentration in the non-Cyborg EcN cells compared to Cyborg EcN, which show no significant decrease in dsDNA concentration. Data is normalized by Day 1 DNA concentration.

Fig. S6A

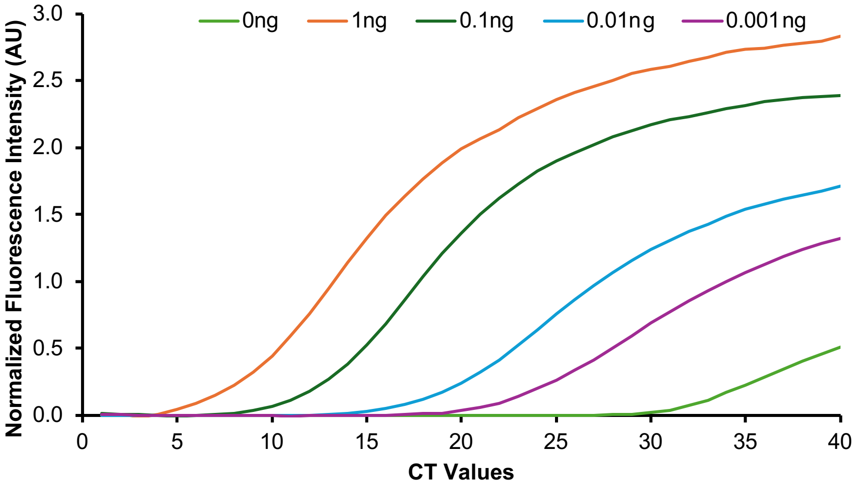

Fig. S6B

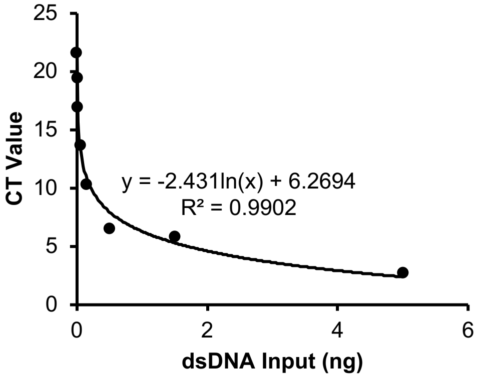

Fig. S6C

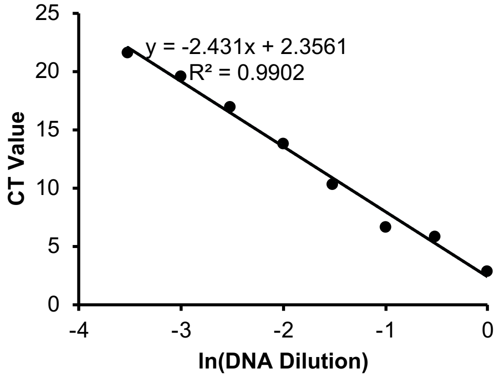

Fig S6: Dilutions and standard curve generation to quantify the synthetic dsDNA in Cyborg Bacterial Cells.

A) Standard curve of 1:10, 1:100 and 1:1000-fold dilutions from full-acrydite modified mOrange 300bp sequence with SYBR Green qPCR Master Mix. The dsDNA was amplified from isolated plasmid DNA and purified before performing three serial dilutions. qPCR amplification curves indicate the decreasing concentration of target mOrange 300bp dsDNA corresponding to the dilutions.

B) Plot of CT-Value versus DNA input per 10 µL reaction mix. dsDNA amplification curves from Fig S6A provided CT values, which were plotted against log dsDNA input concentration. The relationship was linear, with an R^2 value of 0.9902.

C) Plot of CT-Value versus Log-fold DNA Dilution. CT values were obtained from dsDNA amplification curves from Fig S6A and plotted against log-fold DNA dilution. The resulting standard curve was used to approximate DNA concentration in cell lysates.

Supplementary Table 1: Table summarizing the *E. coli* strains used in the study.

**Table 1. *Escherichia coli* Strains**			
**Strain Name**	Characteristic	Designation	Source(s)
Nissle 1917	*Non-Pathogenic, probiotic Escherichia coli* serotype O6:K5:H1	*EcN*	Cress, B. F., 2013
***EcN* Cyborg + dsDNA-PEG hydrogel**	*EcN* Cyborg crosslinked with 5' and 3' modified oligonucleotides not including T7 UTR	*EcN* dsDNA-PEG	This study
Top 10 F'	*E. coli Top 10 F* derivative (mcrA, mcrBC, and mrr mutant) with high transformation efficiency and plasmid production	*Top 10*	Kieffer, N., 2019
***Top 10 F'* Cyborg + dsDNA-PEG hydrogel**	*Top 10* Cyborg crosslinked with 5' and 3' modified oligonucleotides not including T7 UTR	*Top 10* dsDNA-PEG	This Study
***E. coli BL21* (DE3)**	*E. coli B* derivative containing DE3 prophage for T7 RNAP expression under LacUV5 promoter	*BL21*	Daegelen, P., 2009
***BL21*-pET15b mCherry**	High expression mCherry fluorescent bacterial strain with high transformation efficiency	*BL21* mCherry	Shin, E., 2019

***BL21*-pET15b mCherry Cyborg + Full-Acrydite Non-targeting dsDNA-PEG hydrogel**	*BL21* mCherry reporter Cyborg crosslinked with 5' and 3' modified oligonucleotides not including T7 promoter UTR	*BL21* Full-Acrydite Non-targeting	This study
***BL21*-pET15b mCherry Cyborg + Partial-Acyrdite Non-Targeting dsDNA-PEG hydrogel**	*BL21* mCherry reporter Cyborg crosslinked with 5' only modified oligonucleotides not including T7 promoter UTR	*BL21* Partial-Acyrdite Non-Targeting	This study
***BL21*-pET15b mCherry Cyborg + Full-Acrydite Targeting dsDNA-PEG hydrogel**	*BL21* mCherry reporter Cyborg crosslinked with 5' and 3' modified oligonucleotides containing T7 promoter UTR	BL21 Full-Acrydite Targeting	This study
***BL21*-pET15b mCherry Cyborg + Partial-Acrydite Targeting dsDNA-PEG hydrogel**	*BL21* mCherry reporter Cyborg crosslinked with 5' only modified oligonucleotides not including T7 promoter UTR	BL21 Partial-Acrydite Targeting	This study

Supplementary Table 2: Table summarizing the primer sequences.

Table 2. PCR Primers

**DNA Amplicon**	**Primer Name**	**Sequence**	**Size (bp)**
Targeting Sequence	T7 F	TAATACGACTCACTATAGGGAGAC	323
	T7 R	CTTCAGCTTGGCGGTC	
Non-Targeting Sequence	NC F	CCTATGAAGGCTTTCAAACGG	299
	NC R	GCTAGTTATTGCTCAGCGG	
	T7 F-AC	/5Acryd/TAATACGACTCACTATAGGGAGAC	323

Coding DNA Acrydite-Modified	T7 R-AC	/5Acryd/CTTCAGCTTGGCGGTC	
Non Coding DNA Acrydite-Modified	NC F-AC	/5Acryd/CCTATGAAGGCTTTCAAACGG	299
	NC R-AC	/5Acryd/GCTAGTTATTGCTCAGCGG	
50bp DNA	50bp F	/5Acryd/TAATACGACTCACTATAGTACTGTAA	47
	50bp R	/5Acryd/ACAGTGATAGGGGTTTAACAG	
100bp DNA	NC F-AC	/5Acryd/CCTATGAAGGCTTTCAAACGG	121
	100bp R	/5Acryd/GCAGGATGCTTAACATAGGC	

Supplementary Table 3: Table summarizing the qPCR amplification results and amplified DNA concentration amount.

Table 3. qPCR Amplification Results

Strain Name	CT Mean (n=3)	CT Standard Deviation (n=3)	DNA Concentration (pg)
**Top 10 F'**	**20.9**	**0.586**	**2.37**
**Top 10 F' (Transformed only)**	**13.9**	**1.69**	**42.9**

**Top 10 F' (DNA-Hydrogel)**	**13.5**	**1.26**	**52.1**
**EcN**	**21.8**	**1.92**	**1.63**
**EcN (Transformed only)**	**14.2**	**0.218**	**39.1**
**EcN (DNA-Hydrogel)**	**15.9**	**1.982**	**18.5**
